# Aggrecan immobilizes to perineuronal nets through hyaluronan-dependent and hyaluronan-independent binding activities

**DOI:** 10.1016/j.jbc.2025.108525

**Published:** 2025-04-22

**Authors:** Matthew Y. Otsuka, Leslie B. Essel, Ashis Sinha, Gabrielle Nickerson, Seth M. Mejia, Ashley Edge, Russell T. Matthews, Samuel Bouyain

**Affiliations:** 1 Division of Biological and Biomedical Systems, School of Science and Engineering, University of Missouri-Kansas City, Kansas City, Missouri, USA; 2 Department of Neuroscience and Physiology, State University of New York Upstate Medical University, Syracuse, New York, USA

**Keywords:** extracellular matrix, proteoglycan, aggrecan, glycosaminoglycan, hyaluronan, cartilage, perineuronal net, plasticity, protein–carbohydrate interaction, X-ray crystallography

## Abstract

Aggrecan (ACAN) is a large, secreted chondroitin sulfate proteoglycan that includes three globular regions named G1, G2, G3, and is decorated with multiple glycosaminoglycan attachments between its G2 and G3 domains. The N-terminal G1 region interacts with the glycosaminoglycan hyaluronan (HA), which is an essential component of the vertebrate extracellular matrix. In the central nervous system, ACAN is found in perineuronal nets (PNNs), honeycomb-like structures that localize to the surface of parvalbumin-positive neurons in specific neural circuits. PNNs regulate the plasticity of the central nervous system, and it is believed that association between ACAN and HA is a foundational event in the assembly of these reticular structures. Here, we report the cocrystal structure of the G1 region of ACAN in the absence and presence of a HA decasaccharide and analyze the importance of the HA-binding activity of ACAN for its integration into PNNs. We demonstrate that the single immunoglobulin domain and the two Link modules that comprise the G1 region form a single structural unit, and that HA is clamped inside a groove that spans the length of the tandem Link domains. Introducing point mutations in the glycosaminoglycan-binding site eliminates HA-binding activity in ACAN, but, surprisingly, only decreases the integration of ACAN into PNNs. Thus, these results suggest that ACAN can be recruited into PNNs independently of its HA-binding activity.

The glycosaminoglycan hyaluronan (HA) is an abundant component of vertebrate extracellular matrices (ECMs) (1). Unlike other linear, negatively charged, polysaccharides such as heparan sulfate or chondroitin sulfate, HA is neither sulfated nor covalently attached to proteins secreted in the extracellular environment or found at the cell membrane. Instead, HA is assembled from the monosaccharides N-acetylglucosamine (GlcNAc) and glucuronic acid (GlcUA) found in the cytoplasm by HA synthases (HASs). Humans express three HASs named HAS1-3, and these channel-like proteins embedded in the plasma membrane translocate HA chains composed of repeating units of the disaccharide β-1,3-GlcNAc-β-1,4-GlcUA chains into the extracellular environment. Extruded chains of HA may exceed several megadaltons in molecular weight (2). The simple structure of HA perhaps belies the manifold essential functions it has throughout the lifetime of an individual, from mediating morphogenesis in embryos to the scaffolding of the ECM of numerous tissues, including cartilage and the brain, during adulthood (3, 4, 5, 6).

The diverse physiological functions of HA are linked, on the one hand, to its unique water-retention and physicochemical properties and, on the other hand, to its interactions with cell surface receptors or ECM proteins. Several of these HA-binding proteins include one or more ∼100 amino acid repeats called Link modules that were initially identified in two ECM proteins called aggrecan (ACAN) and HA and proteoglycan link protein 1 (HAPLN1, also called link protein or LP) found in porcine cartilage (7, 8). *Hapln1-* or *Acan-*KO mice die soon after birth and present defects in the development of cartilage and bone, underlining the importance of ACAN and HAPLN1 in normal mammalian development (9, 10, 11). ACAN is a large, secreted glycoprotein that spans more than 2500 amino acids in humans (12). Its N-terminus includes a globular region named G1 that shares ∼39% amino acid identity with HAPLN1 and is composed of an immunoglobulin (Ig) domain and two Link modules (Fig. 1*A*) (13). The G1 region of ACAN as well as HAPLN1 bind to HA through their Link modules (14, 15) and biophysical analyses suggest that the N-terminus of ACAN forms a ternary complex with HAPLN1 and HA (16), possibly explaining why *Hapln1-* or *Acan-*KO mice exhibit similar defects in cartilage.

**Figure 1:**
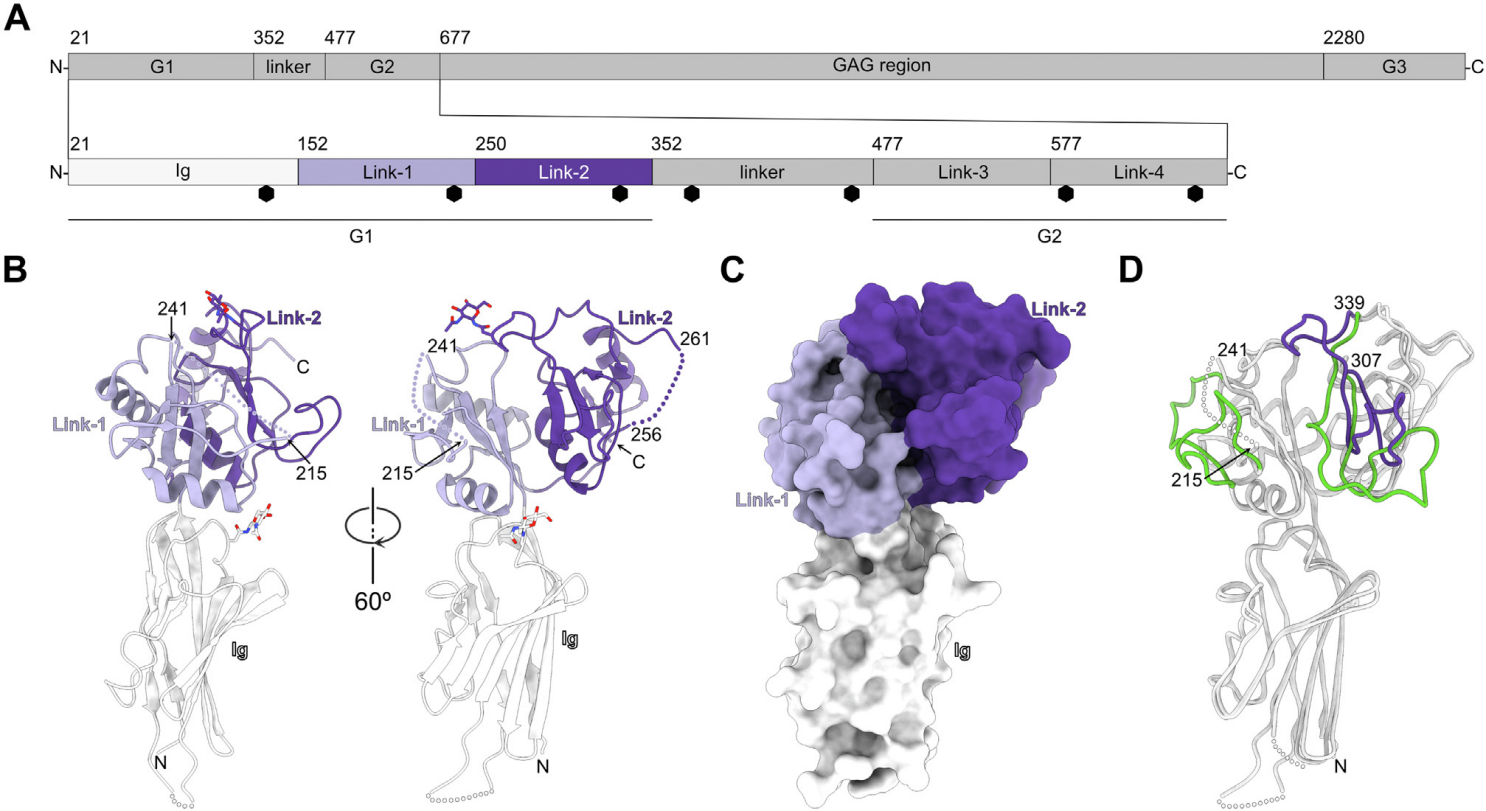
**The crystal structure of the G1 domain of ACAN.***A*, overview of the domain organization of human ACAN. ACAN includes three globular regions named G1, G2, and G3. A large ∼1600 amino acid segment between the G2 and G3 domains includes attachment sites for sulfated glycosaminoglycans (GAGs), such as chondroitin sulfate. The G1-G2 segment includes an N-terminal immunoglobulin (Ig) domain and four Link modules. The G1 region consists of the Ig, Link-1, and Link-2 domains, while the G2 region consists of the Link-3 and Link-4 domains. The Ig, Link-1, and Link-2 are colored *white*, *lilac*, and *violet*, respectively. This color coding is used in *panels B* and *C* below. Amino acid numbering corresponds to human ACAN (UniProt ID# P16112). *Black hexagons* denote predicted N-linked glycosylation sites. *B*, the G1 region of human ACAN is shown in a *ribbon diagram* in two distinct orientations related by a 60° rotation. The Ig, Link-1, and Link-2 domains in chain B of ACAN are colored *white, lilac*, and *violet*, respectively. The letters N and C indicate the N- and C-termini, respectively. Asparagine-linked N-acetylglucosamine residues are shown as *sticks* along with linked asparagine side chains. Three disordered regions in the Ig, Link-1, and Link-2 domains are shown as *dotted lines*. *C*, surface representation of the G1 domain of ACAN. Domains are colored as described in *panel B*. ACAN(G1) is shown in the same orientation as the *right view* in *panel B*. *D*, overlay of the structures of the two chains of ACAN(G1) in the asymmetric unit of ACAN(G1) crystals showing distinct conformations for chains A and B. The two chains are shown as coils and colored *white*. Residues 216 to 240 are not visible in chain B but are well ordered in chain A and shown in *green*. Residues 307 to 339 in chain A are colored *green*, while the same region in chain B is colored *violet*. ACAN, aggrecan.

The physiological functions of ACAN and HAPLN1 are not limited to bone and cartilage, however. In the central nervous system, these two proteins are essential components, along with HA, of a condensed form of ECM called perineuronal nets (PNNs) that form around a specific subset of neurons (17, 18, 19, 20). Broadly, the role of PNNs is to limit neuroplasticity, the ability of the brain to alter its networks as a result of neural activity (21). PNNs are reticular structures that form around the soma and dendrites of subpopulations of neurons throughout the central nervous system. The presence of nets generally correlates with the expression of the Ca^2+^-binding protein parvalbumin and they encase neurons concomitantly with the closing of a period of intense neural remodeling in response to experience called the critical period (22, 23, 24, 25). Although the exact molecular composition of PNNs is thought to vary with the identity of the neural tissue in which they form (26), HA, ACAN, HAPLN1, the matricellular protein tenascin-R (TNR), and the secreted proteoglycan phosphacan appear to be essential for the formation of nets (17, 19, 27, 28). Accordingly, genetic ablation of *Acan*, *Hapln1*, or *Tnr* restores plasticity in mice (17, 19, 29). Crucially, although ACAN is expressed ubiquitously outside of the nervous system, it appears to be found almost exclusively in PNNs in neural tissues (30, 31).

Since (i) HA is abundant in nets (32, 33), (ii) ACAN is an essential component of PNNs that can be released from the surface of net-bearing neurons following treatment with the enzyme hyaluronidase (20, 31, 33), and (iii) the expression of HAS3 in human embryonic kidney cells induces the capture of ACAN in a condensed matrix surrounding these cells (18), it was of interest to determine the extent to which the integration of ACAN into PNNs depends on its physical interaction with HA. In this report, we provide the crystal structures of the G1 region of human ACAN in the absence and presence of a HA oligosaccharide. Mutations of four residues in the glycosaminoglycan-binding site found in the tandem Link domains of ACAN eliminated its HA-binding activity in biolayer interferometry assays. However, in the context of neuronal cultures, these mutations impaired, but did not eliminate the integration of ACAN into PNNs. Overall, these results suggest that ACAN is localized to PNNs through HA-dependent and independent binding activities.

## Results

### The Ig and the two Link modules in the G1 domain of ACAN form a single structural unit

ACAN includes three globular regions named G1, G2, and G3 with a large insert for the attachment of sulfated glycosaminoglycans between the G2 and G3 domains (Fig. 1*A*). The G1-G2 region includes four Link domains arranged in two pairs: the N-terminal Ig domain and the Link-1 and Link-2 pair form the G1 domain that binds to HA (14), while the Link-3 and Link-4 modules form a second pair that corresponds to the G2 region. The crystal structure of the G1 region of ACAN was determined as a first step to investigate the association of ACAN and HA. The structure of deglycosylated ACAN(G1) was solved by molecular replacement using a model generated by AlphaFold (34) and refined to 3.50 Å (R_work_/R_free_ = 0.247/0.284, Table 1, Figs. 1, *B* and *C* and S1). There are two molecules of ACAN in the crystal asymmetric unit. The N-terminal Ig domain of ACAN comprises amino acids L29–K151, while the Link-1 and Link-2 modules span G152–E249 and G250–T350, respectively (Fig. 1*A*). Overall, these three discrete domains form a single structural unit that resembles an inverted letter “L” with dimensions of ∼ 60 × 50 × 20 Å. The interface between the Ig domain and Link-1 buries 430 Å^2^, while the interface between Link-1 and Link-2 buries 682 Å^2^ (Fig. 1, *B* and *C*). The Ig domain does not contact Link-2.

**Table 1:** Data collection and refinement statistics

Crystal	ACAN(G1)	ACAN(G1)–HA_10_ complex
Data collection		
Beamline	APS 22-ID	APS 22-ID
Wavelength (Å)	1.00	1.00
Number of unique reflections	20,877 (4880)	29,486 (2940)
Resolution (Å)	85.91–3.50	50.00–2.58
	(3.83–3.50)	(2.67–2.58)
Space group	P4_1_2_1_2	P2_1_
Unit cell		
a, b, c (Å)	121.49, 121.49, 213.37	73.07, 64.94, 112.21
α, β, γ (°)	90.00, 90.00, 90.00	90.00, 109.33, 90.00
R_merge_	0.125 (1.324)	0.163 (0.542)
R_pim_	0.044 (0.479)	0.104 (0.361)
Completeness (%)	100.0 (100.0)	96.3 (96.9)
Redundancy	8.9 (9.3)	3.2 (2.8)
I/σI	7.5 (2.4)	6.1 (1.5)
CC_1/2_	0.995 (0.683)	0.968 (0.731)
Wilson B factors (Å^2^)	152.5	37.9
Refinement		
PDB code	9DFT	9DFF
Number of protein chains in asymmetric unit	2	2
Resolution (Å)	61.38–3.50	41.03–2.59
	(3.58–3.50)	(2.66–2.59)
Reflections [test]	20,824 [2084]	27,805 [1884]
R_work_/R_free_	0.198/0.245	0.194/0.238
Atoms modeled	4758	5327
Protein	4678	4810
Ligand	80	344
Water	0	173
RMSDs		
Ideal bonds (Å)	0.002	0.005
Ideal angles (°)	0.507	0.770
Average B factors (Å^2^)	167.78	45.73
Protein	166.96	45.23
HA_10_	-	45.15
Ligand	216.13	91.62
Water	-	37.88
Ramachandran statistics		
Favored (%)	95.37	96.13
Allowed (%)	4.46	3.53
Outlier (%)	0.17	0.34
Rotamer outlier (%)	0.20	0.00

Values in parentheses apply to the high-resolution shell.

The two protomers in the asymmetric unit superimpose with an RMSD of 1.25 Å over 251 Cα pairs. However, examination of the two protein chains suggests that there are significant differences in the arrangement of loop regions in the Link domains between the two protomers (Fig. 1*D*). Specifically, although continuous electron density in chain A made it possible to build the entire Link tandem repeats (Fig. S1*A*), two segments in the Link domains of chain B could not be built because of missing or uninterpretable electron density (Fig. 1*B*). These segments include amino acids 216 to 240 and 257 to 260. Furthermore, the conformation of the region 307 to 339 in chain A differs significantly from the one adopted in chain B (Fig. 1*D*). The conformation of this segment in chain A may be an artifact of crystallization because the single GlcNAc attached at N333 is wedged between the Link-1 and Link-2 domains (Fig. S1, *A* and *C*). This monosaccharide residue remained attached to ACAN(G1) after treatment with endoglycosidase H prior to crystallization. In a nondeglycosylated protein, however, the bulk of the asparagine-linked complex carbohydrate would likely prevent such positioning, suggesting that the conformation of the region 307 to 339 in chain A may not reflect its conformation in solution. In addition, amino acids 312 to 330 in chain A protrude away from the Link-2 domain to participate in crystal contact with two symmetry-related mates (Fig. S1*A*). As such, it appears likely that the conformation adopted by chain B is more physiologically relevant than the one adopted by chain A.

### The two Link domains assemble a contiguous binding site that accommodates a single HA decasaccharide

**Figure 2:**
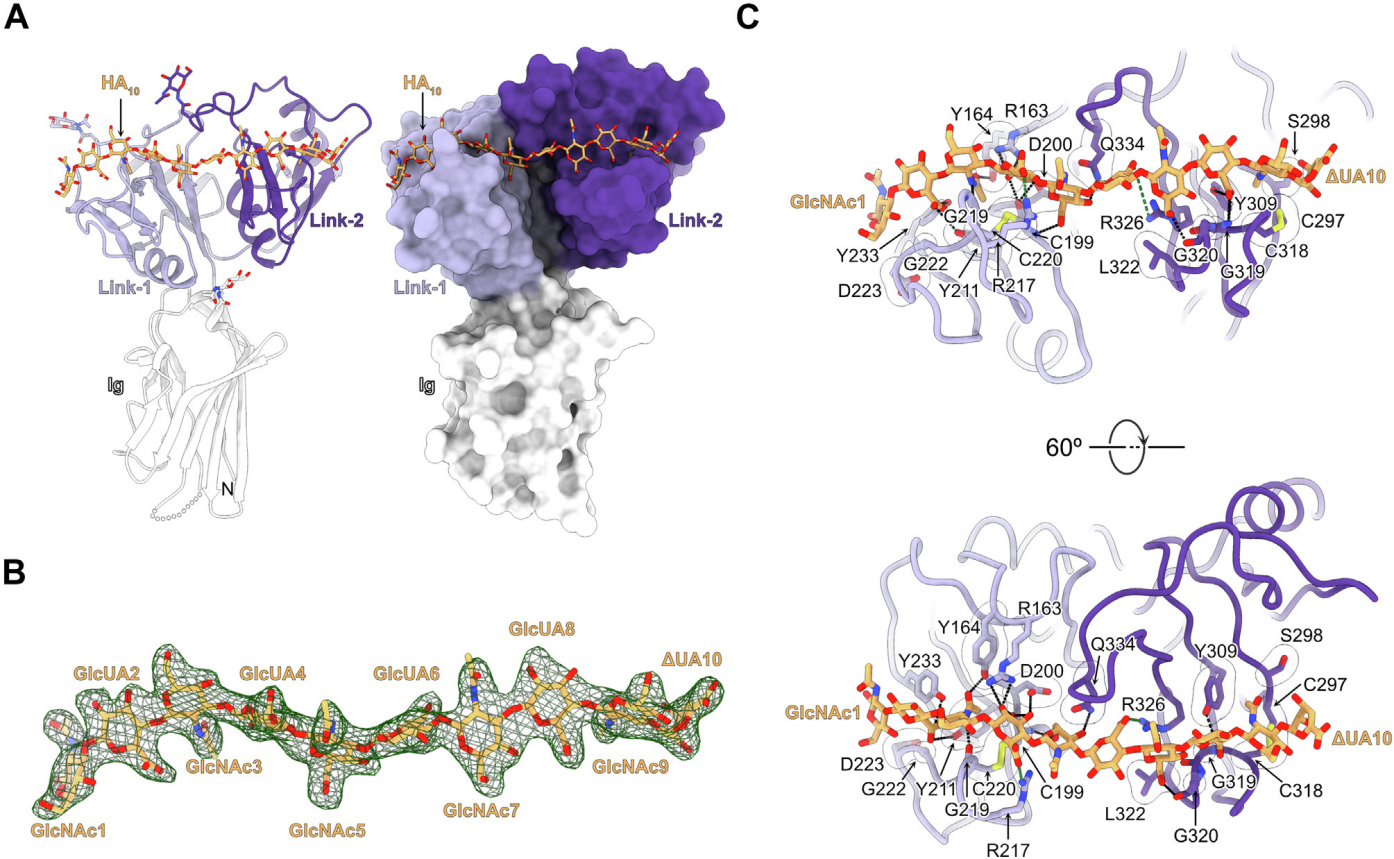
**Crystal structure of the complex between ACAN and a HA oligosaccharide.***A*, the G1 domain of ACAN bound to HA_10_ is shown as a *ribbon diagram* on the *left* and in *surface representation* on the *right*. The bound HA decasaccharide is shown as *orange sticks* in both views. The Ig, Link-1, and Link-2 domains in ACAN are colored *white, lilac*, and *violet*, respectively. The letters N and C indicate the N- and C-termini, respectively. Asparagine-linked N-acetylglucosamine residues are shown as *sticks* along with linked asparagine side chains. *B*, mFo-DFc electron density map for the bound HA_10_ calculated by omitting the oligosaccharide from the model is contoured at 3 σ and shown as a *green mesh*. The oligosaccharide is in the same orientation as the one shown in *panel A*. Residues in the HA oligosaccharide are colored *orange*. The terminal residue, 4-deoxy-alpha-L-threo-hex-4-enopyranuronic acid, created during the preparation of the HA oligosaccharide, is labeled ΔUA. *C*, detailed view of the interactions between HA_10_ and residues in the Link-1 and Link-2 of ACAN(G1). The *top view* is in the same orientation as the view shown in *panel A*. The bound HA is shown as *orange sticks*. Link-1 and Link-2 are shown as *coils*. Contacting protein residues are represented as *sticks*, while *transparent surfaces* denote residues involved in van der Waals or packing interactions. Potential hydrogen bonds are represented as *black dashed lines* between interacting atoms, while salt bridge interactions involving R217 and R326 are shown as *green dashed lines*. Side chains or main chain atoms in ACAN are displayed only if they contact HA_10_. Only the first and tenth residues of HA_10_ are labeled for the sake of clarity. Residues in Link-1 are colored *lilac*, while residues in Link-2 are colored *violet*. ACAN, aggrecan; GlcUA, beta-D-glucopyranuronic acid; HA, hyaluronan; Ig, immunoglobulin; ΔUA, 4-deoxy-alpha-L-threo-hex-4-enopyranuronic acid.

Previous work has indicated that ACAN may associate with HA oligosaccharides that comprises at least 10 monosaccharides (16, 35, 36). Thus, the G1 domain of ACAN was crystallized in the presence of a fivefold molar excess of a HA decasaccharide (HA_10_) to obtain atomic level information between ACAN and HA. The crystal structure of the ACAN(G1)-HA_10_ complex was solved by molecular replacement and refined to 2.59 Å (R_work_/R_free_ = 0.194/0.238, Table 1, Figs. 2, and S2). There are two molecules of ACAN in the asymmetric unit that superimpose with an RMSD of 0.18 Å over 301 shared Cα positions. The electron density for the bound HA_10_ is well-defined for all the sugar residues in the oligosaccharide (Figs. 2*B* and S2). The oligosaccharide buries 820 Å^2^ of surface area and fits within a groove that spans both Link domains in ACAN, while the Ig domain does not make any contact with HA (Fig. 2*A*). Comparison of the bound and free form of ACAN indicates that the segment comprising residues 217 to 241 that was disordered in HA-free ACAN is now ordered and makes substantial contact with the oligosaccharide. Concomitantly, HA_10_ adopts an almost linear conformation that differs from the structure of free HA obtained from powdered diffraction (Fig. S3). This change is consistent with the alteration of the HA structure reported upon binding to ACAN(G1) (37). Finally, comparing the HA-binding site in ACAN and in mouse CD44, the other available complex between a Link module and a HA oligosaccharide (38), shows that the sugar-binding grooves in Link-1 and Link-2 both match the HA-binding site in CD44 (Fig. S4*A*). However, a loop extends from Link-2 to bind to HA between the two Link domains, giving the appearance that the oligosaccharide is pinched at the interface between Link-1 and Link-2. This loop is absent in CD44 and corresponds to an insertion of eight amino acids between residues 109 and 110 of CD44 (Fig. S4*B*).

Overall, the 10 monosaccharide residues in the crystallized HA oligosaccharide contact 18 amino acids in ACAN through van der Waals or hydrogen bonding interactions (Fig. 2*C*). All but three of these eighteen HA-contacting residues appear well conserved among vertebrate ACAN proteins (Fig. S5). In contrast to the interface between CD44 and HA, only one aliphatic amino acid, L322, contacts the bound HA (Fig. S4*B*). Instead, van der Waals contacts occur between the side chains of C199, D200, Y211, D223, C297, S298, Y309, L322, and Q334, on the one hand, and GlcNAc1, GlcNAc3, GlucA4, GlucA6, GlcNAc7, GlucA8, GlcNAc9, and the terminal ΔUA10 on the other hand. Furthermore, sugar residues GlucA2-GlcA8 form an extensive network of eighteen hydrogen bonds with ACAN. These include the main chain atoms of C199, G219, G319, and G320 forming seven hydrogen bonding interactions with GlcNAc3, GlucA4, GlcNAc7, and GlucA8. In addition, the side chain atoms of R163, Y164, D200, Y211, R217, Y233 in Link-1 contact GlucA2 to GlcNAc5, while the side chains of Y309, R326, and Q334 form hydrogen bonds with GlcNAc5 to GlucA8. Surprisingly, there are only two salt bridge interactions between HA and ACAN residues in spite of the five negative charges found in HA. These interactions involve the guanidinium groups of R217 and R326, which contact the carboxylate groups of GlucA4 and GlucA6 at the center of the bound decasaccharide (Fig. 2*C*).

### Mutations in the Link-1 and Link-2 domains of ACAN impair binding to HA

**Figure 3:**
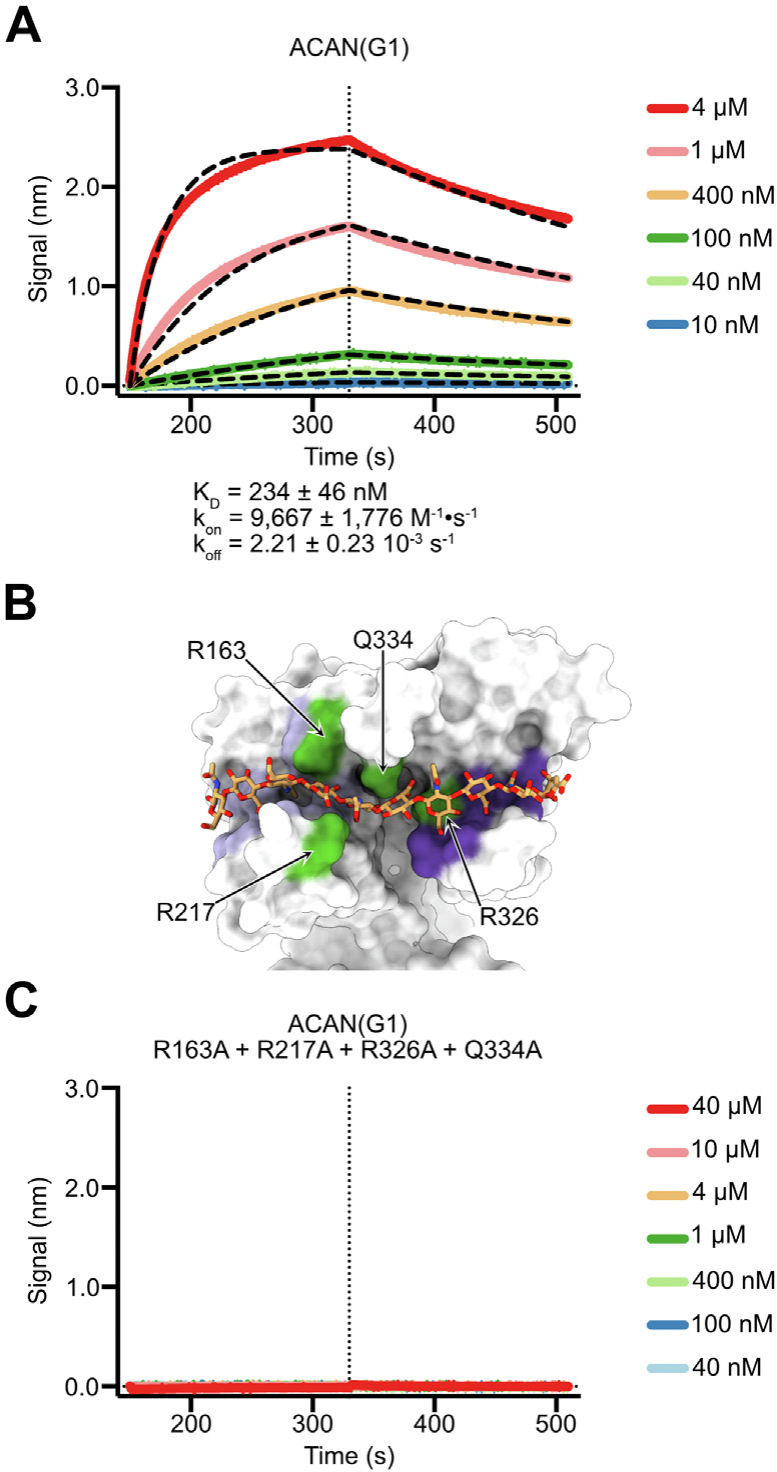
**Characterization of ACAN(G1) binding to immobilized HA by biolayer interferometry.***A*, binding of decreasing concentrations of the G1 domain of human ACAN to 20-kDa HA immobilized onto streptavidin sensors. Representative association and dissociation curves are shown here along with a *vertical dashed line* that indicates the start of the dissociation phase. Raw binding data are shown in distinct colors and were analyzed using a 1:1 binding model. Fitted binding curves are shown as *black dashed lines*. Values for the affinity constant (K_D_), the rate of association (k_on_), and the rate of dissociation (k_off_) are reported as average ± SD from eleven independent experiments using three distinct biological replicates. Additional information about individual experiments used in affinity calculations is listed in Table S1. *B*, the tandem Link modules in ACAN(G1) are shown as a *white surface*, while the bound HA is shown as *orange sticks*. HA-binding residues in the Link-1 module are colored *lilac*, while HA-binding residues in Link-2 are colored *violet*. The positions of residues that are changed to alanine in the HA-binding mutant of ACAN(G1) are colored *green*. This view is in the same orientation as the one shown in the *bottom view* of Figure 2*C*. *C*, binding of decreasing concentrations of the G1 domain of human ACAN including mutations to alanine at positions R163, R217, R326, and Q334 to 20-kDa HA immobilized onto streptavidin sensors. Representative association and dissociation curves are shown here along with a *vertical dashed line* that indicates the start of the dissociation phase. The raw binding data did not show any interaction between immobilized HA and this variant of ACAN(G1). ACAN, aggrecan; HA, hyaluronan.

A binding assay using biolayer interferometry (BLI) was designed to quantify the interaction between HA and ACAN(G1) as well as validate the structural results described above. In these experiments, the binding of increasing concentrations of ACAN(G1) to 20-kDa monobiotinylated HA immobilized on a streptavidin biosensor was measured. The traces were fit to a 1:1 model and the apparent binding affinity (K_D_) was calculated from the rates of association (k_on_) and dissociation (k_off_) (Fig. 3*A*). In these experiments, ACAN binds to immobilized HA with an affinity of 234 nM, which is consistent with the value of 226 nM reported in an earlier study using surface plasmon resonance (14).

With the binding assay validated, four mutations to alanine were introduced at the interface between ACAN(G1) and the bound HA_10_ to confirm that the contacts observed in the crystals reflect interactions in solution. Residues R163, R217, R326, and Q334 were selected because they interact with the trisaccharide GlucA4-GlcNAc5-GlucA6 located centrally in HA_10_ and appear strictly conserved in selected vertebrate species (Figs 2*C*, 3*B*, and S5). The mutant protein behaved comparably with WT ACAN(G1) during purification, specifically size-exclusion chromatography, suggesting that the introduction of the mutations did not introduce gross structural alterations. Furthermore, the structural integrity of the mutant ACAN(G1) was verified by CD spectropolarimetry, which confirmed that the introduction of alanine residues did not alter the secondary structure of ACAN(G1) (Fig. S6). In BLI assays, the mutant ACAN(G1) did not associate with immobilized HA (Fig. 3*C*). To confirm that the interaction of ACAN(G1) with the large chains of HA found in PNNs is consistent with the experiments carried out with 20-kDa HA chains, BLI assays were repeated with 1-MDa monobiotinylated HA (Fig. S6). In these BLI experiments, the measured K_D_ was not statistically different from the one measured for the 20-kDa HA (Fig. S7, *A* and *B*). Finally, the mutant of ACAN(G1) did not associate with the larger HA chains, consistent with the results of Figures 3*C* and S7*C*). Overall, these results indicate that the protein-HA interface identified in the complex structure likely represents the arrangement of ACAN and HA in the matrix.

**Figure 4:**
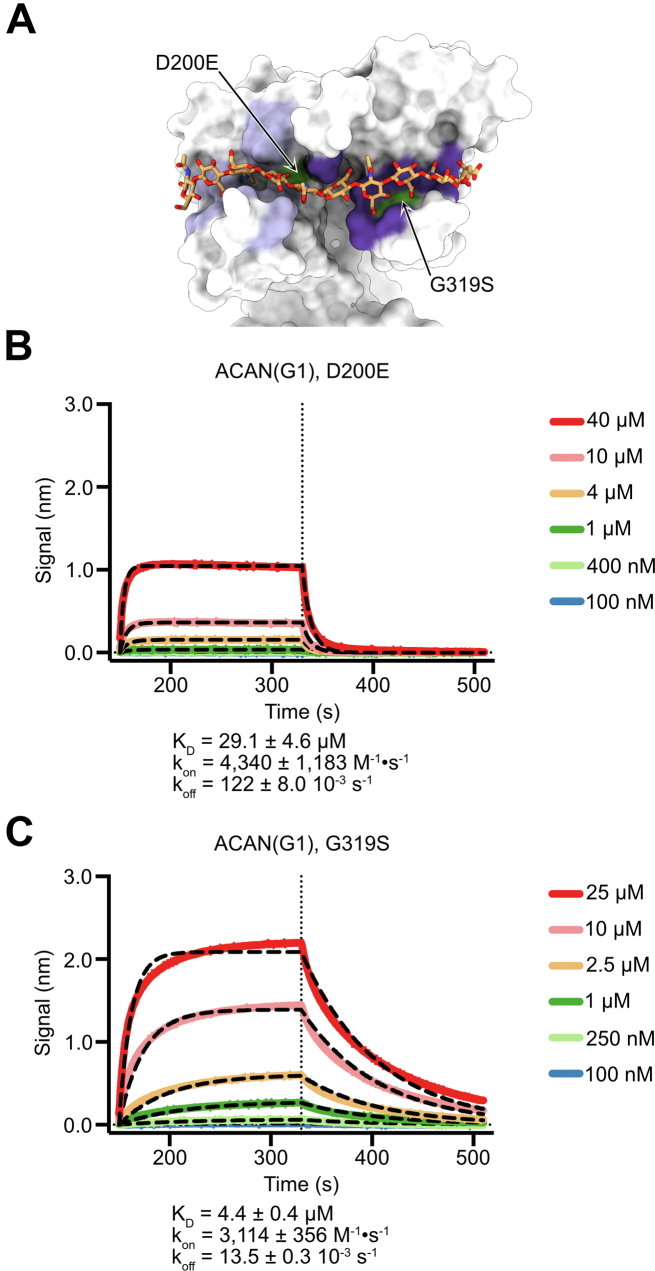
**Characterization of missense mutations found in the HA-binding site of ACAN(G1) associated with osteochondritis dissecans.***A*, the tandem Link modules in ACAN(G1) are shown as a *white surface*, while the bound HA is shown as *orange sticks*. HA-binding residues in the Link-1 module are colored *lilac*, while HA-binding residues in Link-2 are colored *violet*. The positions of the two missense mutations in the HA-binding site associated with osteochondritis dissecans are colored *green*. This view is in the same orientation as the one shown in the *bottom view* of Figure 2*C*. *B*, binding of decreasing concentrations of the G1 domain of human ACAN including the change D200E to 20-kDa HA immobilized onto streptavidin sensors. Representative association and dissociation curves are shown here along with a *vertical dashed line* that indicates the start of the dissociation phase. Raw binding data are shown in distinct colors and were analyzed using a 1:1 binding model. Fitted binding curves are shown as *black dashed lines*. Values for the affinity constant (K_D_), the rate of association (k_on_), and the rate of dissociation (k_off_) are reported as average ± SD from five independent experiments using two distinct biological replicates. Additional information about individual experiments used in affinity calculations is listed in Table S1. *C*, binding of decreasing concentrations of the G1 domain of human ACAN including the change G319S to 20-kDa HA immobilized onto streptavidin sensors. Representative association and dissociation curves are shown here along with a *vertical dashed line* that indicates the start of the dissociation phase. Raw binding data are shown in distinct colors and were analyzed using a 1:1 binding model. Fitted binding curves are shown as *black dashed lines*. Values for the affinity constant (K_D_), the rate of association (k_on_), and the rate of dissociation (k_off_) are reported as average ± SD from five independent experiments using two distinct biological replicates. Additional information about individual experiments used in affinity calculations is listed in Table S1. ACAN, aggrecan; HA, hyaluronan.

Mutations in ACAN have been linked to several human diseases that affect bone and/or cartilage (39, 40). Thus, it was of interest to examine whether reported mutations map to the HA-binding site and what their effect on HA binding might be. We searched the ClinVar database for missense mutation in ACAN and identified two changes in residues that contact HA in the crystal structure (Fig. 2*C*) (41). These residues also appear conserved in the G1 domains of selected vertebrate species (Fig. S5). These two mutations, a change from aspartate to glutamate at position 200 (D200E) in Link-1 and a change from glycine to serine at position 319 (G319S) in Link-2 are associated with osteochondritis dissecans, a condition that affects bone and cartilage (Fig. 4*A*) (42). Variants of ACAN(G1) including these changes were expressed and their HA-binding properties assessed using BLI (Fig. 4). Introducing the amino acid changes did not appear to introduce any gross alteration of the G1 domain, as the mutant proteins could be purified to homogeneity from conditioned media as easily as the WT protein.

Although changes from aspartate to glutamate are often considered benign, introducing this mutation at position 200 decreased the affinity of ACAN(G1) for HA more than a 100-fold (29.1 μM *versus* 234 nM, Fig. 4*B*). The carboxylate group of D200 forms a hydrogen bond with a hydroxyl group in GlucA4 and the introduction of an additional methyl group in the side chain might put the carboxylate group too far for such an interaction. In addition, D200 lies deep in the HA-binding crevice so that elongation of the side chain after the change to glutamate might push the HA chain away from the binding site because of a reduction in the size of the HA-binding groove. The change at position 319 from a glycine to a serine does not impact the affinity for HA to the same extent as D200E, but still leads to a ∼20-fold reduction in affinity (4.4 μM *versus* 234 nM). The main chain nitrogen atom of G319 forms a hydrogen bond with residue GlucA8 and this interaction would not be expected to change upon mutating the sidechain to serine. However, the Cα atom of G319 is located 4.1 Å from the Cγ1 atom of I311. Upon mutation of G319 to a serine, the Cβ of S319 would be located 3 Å and 3.2 Å from the Cγ1 and Cγ2 atoms of I311, respectively, leading to potential steric clashes. Thus, the decrease in affinity upon introducing the G319S change might be explained by distortion in the HA-binding site caused by the bulkier serine residue. It is beyond the scope of the present study to determine whether defective anchoring to HA stemming from mutations in the binding site cause osteochondritis dissecans, but the availability of the ACAN-HA structure may now provide an opportunity to interpret the effect of ACAN mutations found in human patients.

### The G2 domain of ACAN does not bind to HA in spite of residue conservation in the HA-binding site

**Figure 5:**
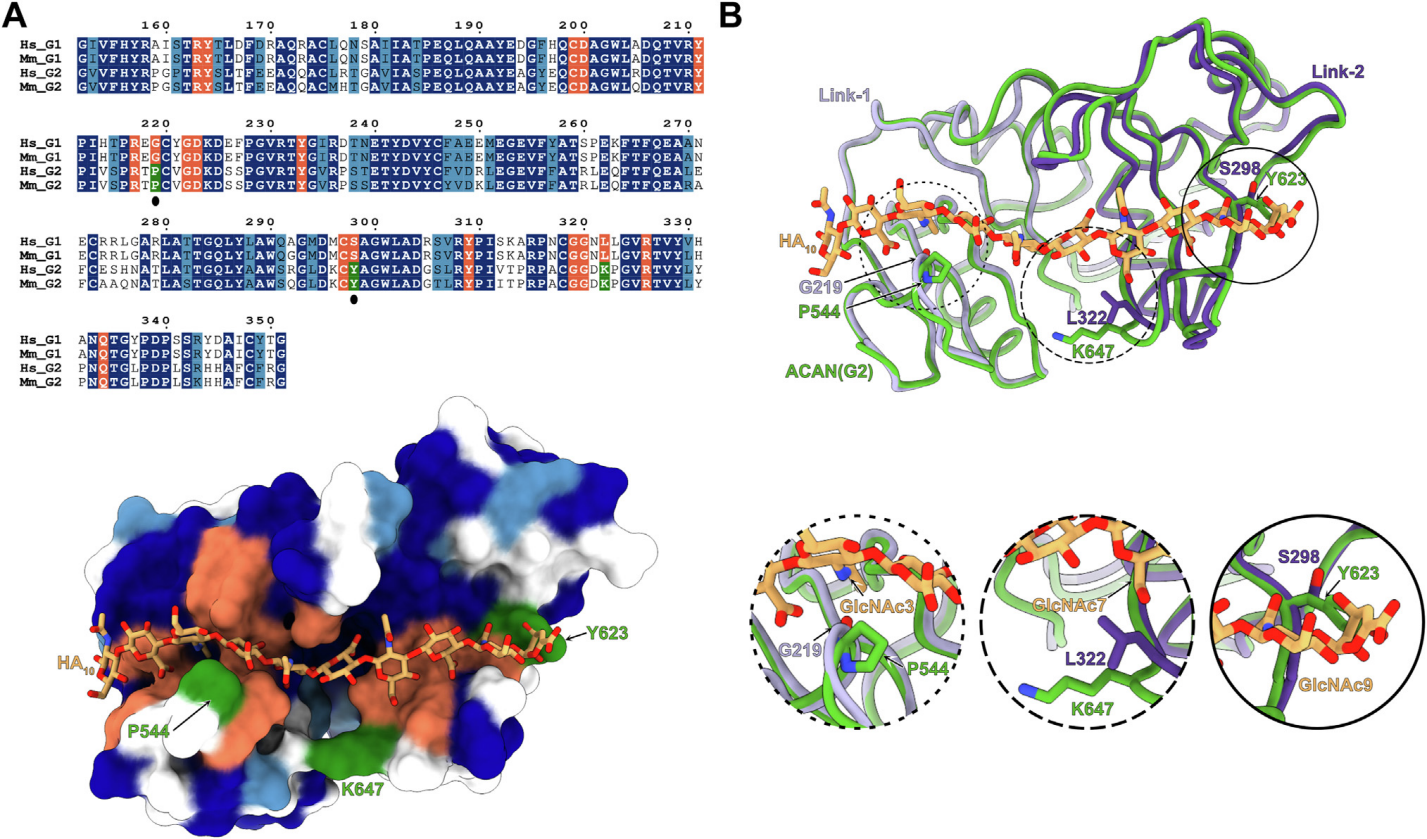
**Comparison of the** L**ink modules in the G1 and G2 regions of ACAN.***A*, amino acid conservation between the tandem Link modules found in the G1 and G2 regions of human and mouse ACAN with amino acid numbers corresponding to human ACAN(G1). Identical residues are shaded in *dark blue*, while similar residues are shaded in *blue*. Residues that interact with HA in human ACAN are shaded coral. HA-binding residues that are not conserved in the G2 region are shaded *green*. *Black dots* under G219 and S298 indicate HA-binding residues that are distinct between the G1 and G2 domains and are mutated in the experiments shown in Figure 6*D*. The *bottom view* shows a surface representation of the model of ACAN(G2) with the oligosaccharide bound in the G1 domain of ACAN. The surface of ACAN(G2) is colored according to residue conservation in the sequence alignment. *B*, overlay of the structures of the tandem Link modules in ACAN(G1) and in ACAN(G2). The model for ACAN(G2) was generated using AlphaFold3. Proteins are shown in *coil representation*, while the bound oligosaccharide is shown as *orange sticks*. The Link-1 and Link-2 domains in ACAN(G1) are colored *lilac* and *violet*, respectively, while the Link-3 and Link-4 modules in ACAN(G2) are colored *green*. The three HA-binding residues in ACAN(G1) that are distinct in ACAN(G2) are shown as *sticks* and colored *lilac* or *violet*. Insets show detailed views of these three residues. The corresponding residues in ACAN(G2) are shown as *green sticks*. ACAN, aggrecan; HA, hyaluronan. Hs, *Homo sapiens*; Mm, *Mus musculus*.

With the ACAN-HA structure determined, it was of interest to examine the conservation of HA-binding residues in the tandem Link domains found in the G2 region of ACAN. In humans, these domains share 65% amino acid identity with those found in the G1 domains (Fig. 5*A*). Furthermore, comparison of the Link-1 and Link-2 domains of ACAN(G1) with a model of the G2 domain generated by AlphaFold3 indicates a high degree of structural similarity, as the two regions superimpose with an RMSD of 0.72 Å over 199 Cα atoms (Fig. 5*B*). Examination of the conservation of HA-binding residues indicates that 15 of 18 residues that contact HA in G1 are conserved in G2. The only changes, G219P, S298Y, and L322K in humans, correspond to residues that contact GlcNAc3, GlcNAc9, and GlcNAc7, respectively, in the crystal structure (Figs. 2*C* and 5*B*).

**Figure 6:**
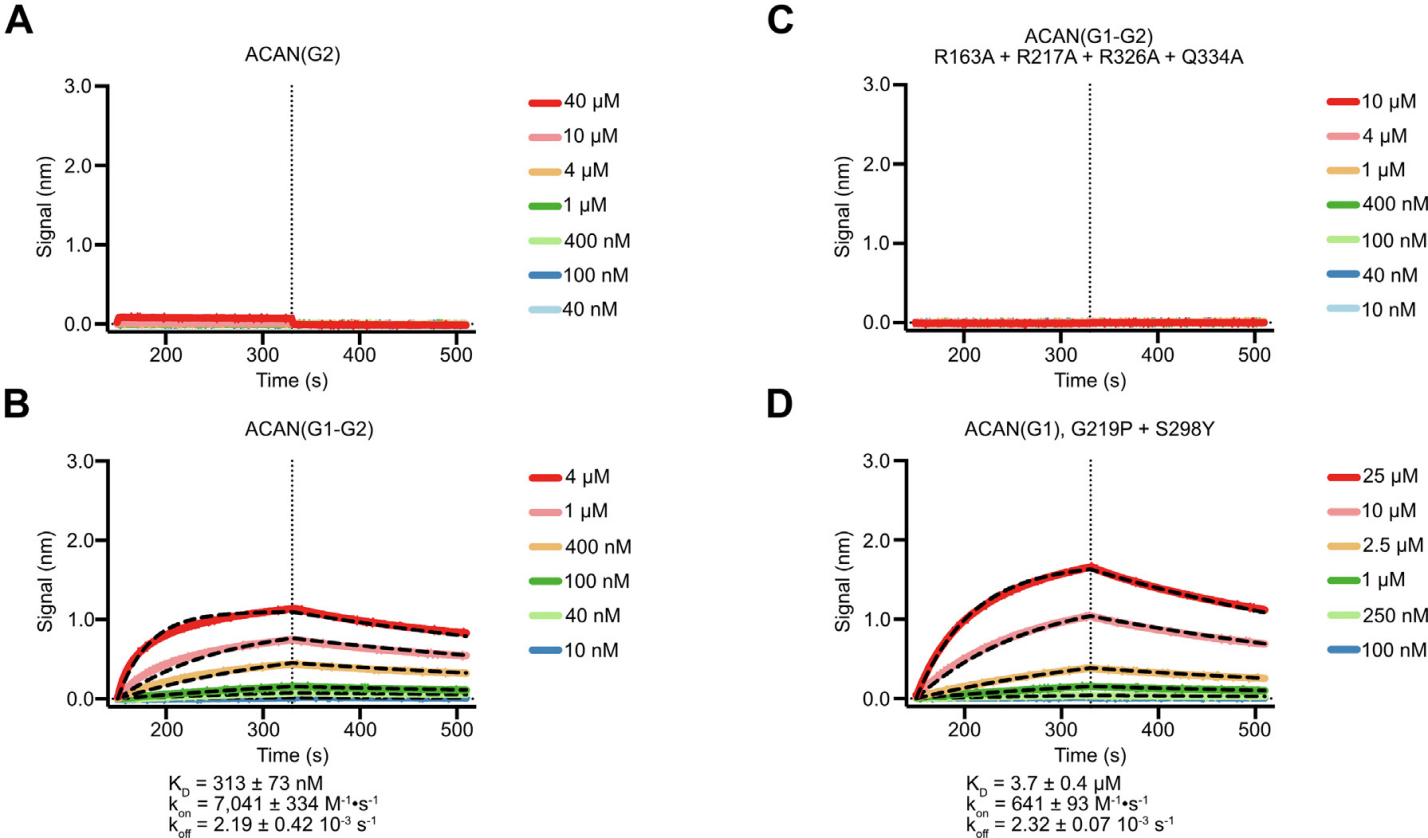
**The tandem** L**ink modules in the G2 domain of ACAN do not bind HA.***A*, binding of decreasing concentrations of the G2 domain of human ACAN to 20-kDa HA immobilized onto streptavidin sensors. Representative association and dissociation curves are shown here along with a *vertical dashed line* that indicates the start of the dissociation phase. The raw binding data did not show any interaction between immobilized HA and ACAN(G2). *B*, binding of decreasing concentrations of the N-terminal G1-G2 region of human ACAN to 20-kDa HA immobilized onto streptavidin sensors. Representative association and dissociation curves are shown here along with a *vertical dashed line* that indicates the start of the dissociation phase. Raw binding data are shown in distinct colors and were analyzed using a 1:1 binding model. Fitted binding curves are shown as *black dashed lines*. Values for the affinity constant (K_D_), the rate of association (k_on_), and the rate of dissociation (k_off_) are reported as average ± SD from seven independent experiments using three distinct biological replicates. Additional information about individual experiments used in affinity calculations is listed in Table S1. *C*, binding of decreasing concentrations of the N-terminal G1-G2 region of human ACAN including mutations to alanine at positions R163, R217, R326, and Q334 to 20-kDa HA immobilized onto streptavidin sensors. Representative association and dissociation curves are shown here along with a *vertical dashed line* that indicates the start of the dissociation phase. The raw binding data did not show any interaction between immobilized HA and this variant of ACAN(G1-G2). *D*, binding of decreasing concentrations of the G1 domain of human ACAN including the changes G219P and S298Y to 20-kDa HA immobilized onto streptavidin sensors. Representative association and dissociation curves are shown here along with a vertical dashed line that indicates the start of the dissociation phase. Raw binding data are shown in distinct colors and were analyzed using a 1:1 binding model. Fitted binding curves are shown as *black dashed lines*. Values for the affinity constant (K_D_), the rate of association (k_on_), and the rate of dissociation (k_off_) are reported as average ± SD from four independent experiments using two distinct biological replicates. Additional information about individual experiments used in affinity calculations is listed in Table S1. ACAN, aggrecan; HA, hyaluronan.

It was thus interesting that previous reports suggested that the G2 segment does not bind to HA given the high degree of structural and sequence similarity between the Link-1-Link-2 and Link-3-Link-4 pairs (14, 43). However, BLI assays conducted with purified human ACAN(G2) showed no quantifiable interaction with HA, in agreement with prior reports (Fig. 6*A*). Despite this result, we wondered whether the presence of the G2 domain would alter the affinity of the G1-G2 segment of ACAN for HA when compared to the G1 region only. BLI assays were thus carried out using purified ACAN(G1-G2). The K_D_ for the interaction with immobilized HA was measured at 313 nM (Fig. 6*B*). This affinity constant is slightly higher than the 234 nM measured for ACAN(G1). The differences between the values of K_D_ measured for ACAN(G1) and ACAN(G1-G2) are statistically significant and are entirely due to differences in the on-rates, because the off-rates are not statistically different (Fig. S8). Furthermore, introducing alanine residues in place of R163, R217, R326, and Q334 in ACAN(G1-G2) ablated HA-binding activity, as was the case for ACAN(G1) (Figs. 3*C* and 6*C*). As such, our biochemical analyses confirm previous reports that the G2 domain of ACAN does not bind to HA. These results also indicate that the HA-binding activities of the G1 and G1-G2 regions of ACAN are similar.

To provide a rationale for the lack of HA-binding activity in G2, the changes G219P and S298Y were introduced in ACAN(G1) and the variant tested in HA-binding assays using BLI (Fig. 6*E*). We did not introduce the change L322K because the K647 in G2 points away from the HA-binding site and is thus unlikely to interfere with HA binding. The introduction of the G219P and S298Y changes in ACAN(G1) led to a 16-fold decrease in affinity for HA compared to WT (Figs. 3*A* and 6*E*). Interestingly, the association rate constant between HA and the variant was reduced about 10-fold, while the dissociation rate constant is almost identical to WT. These findings suggest that amino acid changes in the HA-binding site contribute partly to the lack of interaction between ACAN(G2) and HA. However, additional structural alterations in G2 compared to G1 that are not currently predicted in the AlphaFold3-generated model may explain the absence of HA-binding affinity in this domain. These may be identified when experimental structural information on G2 becomes available.

### The HA-binding activity of ACAN is important for its integration into PNNs

In addition to being an essential component of the cartilage ECM, ACAN plays a critical role in the structure and function of PNNs, a condensed form of neuronal ECM rich in HA (19). As such, it was of interest to investigate the importance of the HA-binding activity of ACAN to its integration in PNNs. To accomplish this objective, we assessed the binding of ACAN fragments to dissociated cultures of cortical neurons. PNNs can form in such primary neuronal cultures and this model system has been used in the past to investigate the role of phosphacan in their assembly (28, 44). Endogenous ACAN localizes to nets found in cortical cultures, so another component of PNNs, the chondroitin sulfate proteoglycan neurocan (NCAN) was used to mark their presence (25, 45). To differentiate between endogenous ACAN and fragments added to cultures, regions of ACAN were expressed as V5-tagged fusion proteins and purified from the conditioned media of HEK293 cells.

**Figure 7:**
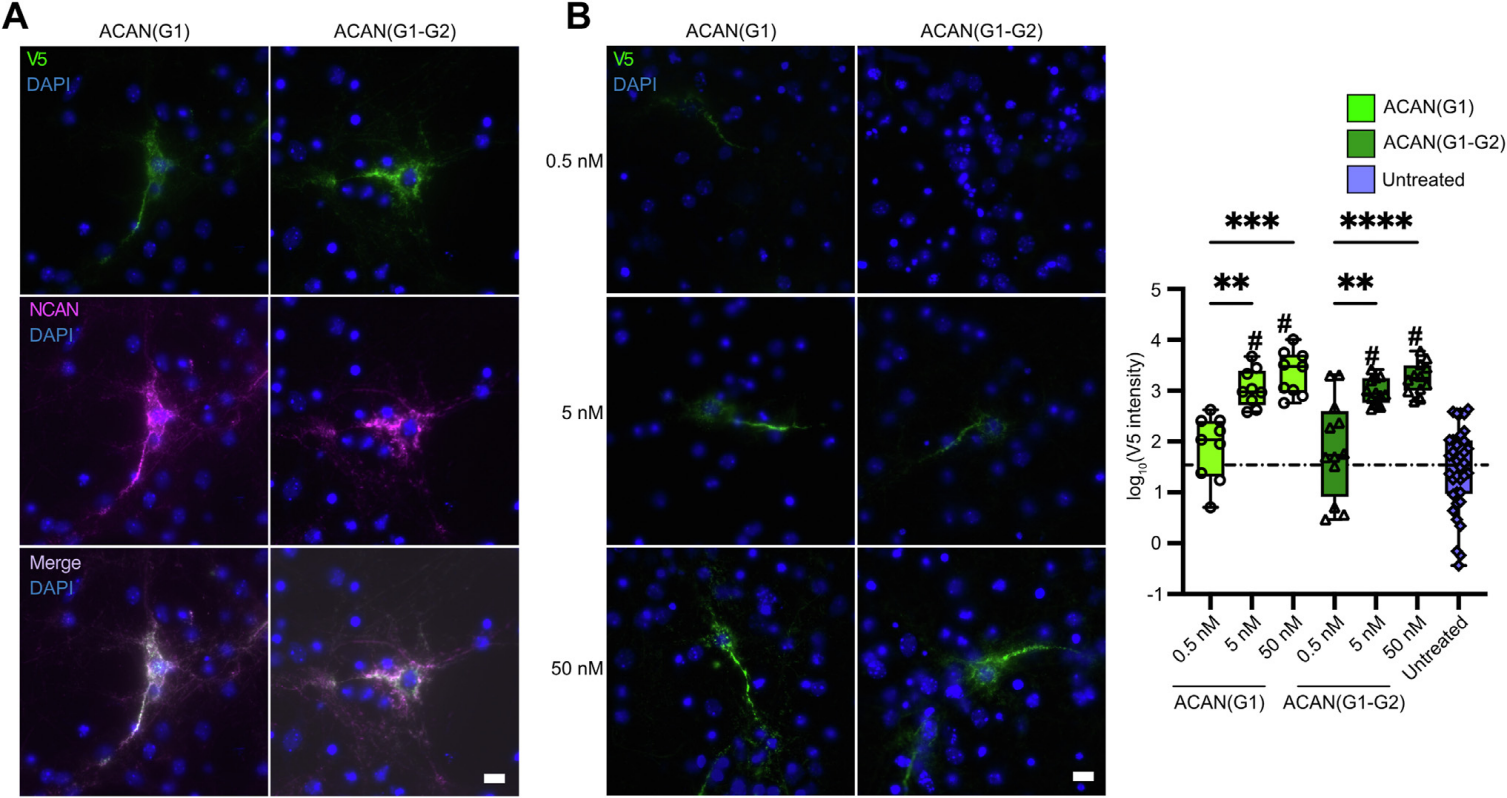
**ACAN(G1) and ACAN(G1-G2) fragments bind specifically to PNNs.***A*, purified V5-tagged ACAN(G1) and ACAN(G1-G2) at 5 nM bound highly and specifically to PNNs as shown by the colocalization of V5 reactivity with the PNN marker NCAN in cultured cortical neurons: V5 colocalization with NCAN reactivity, R = 0.971 ± 0.032 and 0.965 ± 0.037 for ACAN(G1) and ACAN(G1-G2), respectively, by Manders' colocalization coefficient. N = 4 independent cultures, 3 large tiled (1259 μm × 941 μm) images per culture per condition. The scale bar represents 10 μm. *B*, binding of WT ACAN(G1) and ACAN(G1-G2) assessed by V5 reactivity was optimal between 0.5 nM and 50 nM. This range appears ideal for studying the interactions of ACAN fragments with PNNs. Specific and intense PNN binding at 5-nM and 50-nM doses for both ACAN(G1) and ACAN(G1-G2) was observed compared to untreated control cells (ordinary one-way ANOVA, *p* < 0.0001, F (6,92) = 3.807; Tukey’s *post hoc* testing, # indicates *p* < 0.0001 compared to the untreated control group). Cells treated with 5-nM and 50-nM doses also showed significantly greater binding than at 0.5 nM for both ACAN(G1) (5 nM, *p* = 0.0063, 50 nM *p* = 0.0002) and ACAN(G1-G2) (5 nM, *p* = 0.0015, 50 nM *p* < 0.0001). N = 4 independent cultures, 3 large tiled (1259 μm × 941 μm) images per culture per condition. The scale bar represents 10 μm. ACAN, aggrecan; DIV, day *in vitro*; NCAN, neurocan; PNN, perineuronal net.

We first evaluated the binding of the V5-tagged G1 and G1-G2 domains of ACAN to PNNs in primary neuronal cultures (Fig. 7). Interestingly, both fragments of ACAN bound highly and very specifically to PNNs on cultured cortical neurons as evidenced by the similar staining patterns obtained with antibodies against V5 or against the PNN component NCAN. The correlation coefficient R is above 0.95 for the binding of ACAN constructs to endogenous NCAN staining in PNNs (Fig. 7*A*). To assess carefully potential differences in binding affinity or specificity, ACAN fragments were added to cultures in three distinct amounts and concentration curves were generated (Fig. 7*B*). In these experiments, we determined that exogenous addition of constructs at concentrations between 0.5 nM and 50 nM represented the ideal range to study interactions of ACAN fragments with net-bearing neurons. Little binding was detected at concentrations below 0.5 nM, while there was little change in PNN binding intensity but more nonspecific binding at concentrations above 50 nM (Fig. S9). Significant binding compared to untreated cells was measured at both 5 nM and 50 nM for both the G1 and G1-G2 fragments (Fig. 7*B*, ACAN(G1): 5 nM *p* = 0.0063, 50 nM *p* = 0.0002; ACAN(G1-G2): 5 nM *p* = 0.0015, 50 nM *p* < 0.0001). In these experiments, the association of the G1 and G1-G2 regions to net-bearing neurons was not significantly different.

**Figure 8:**
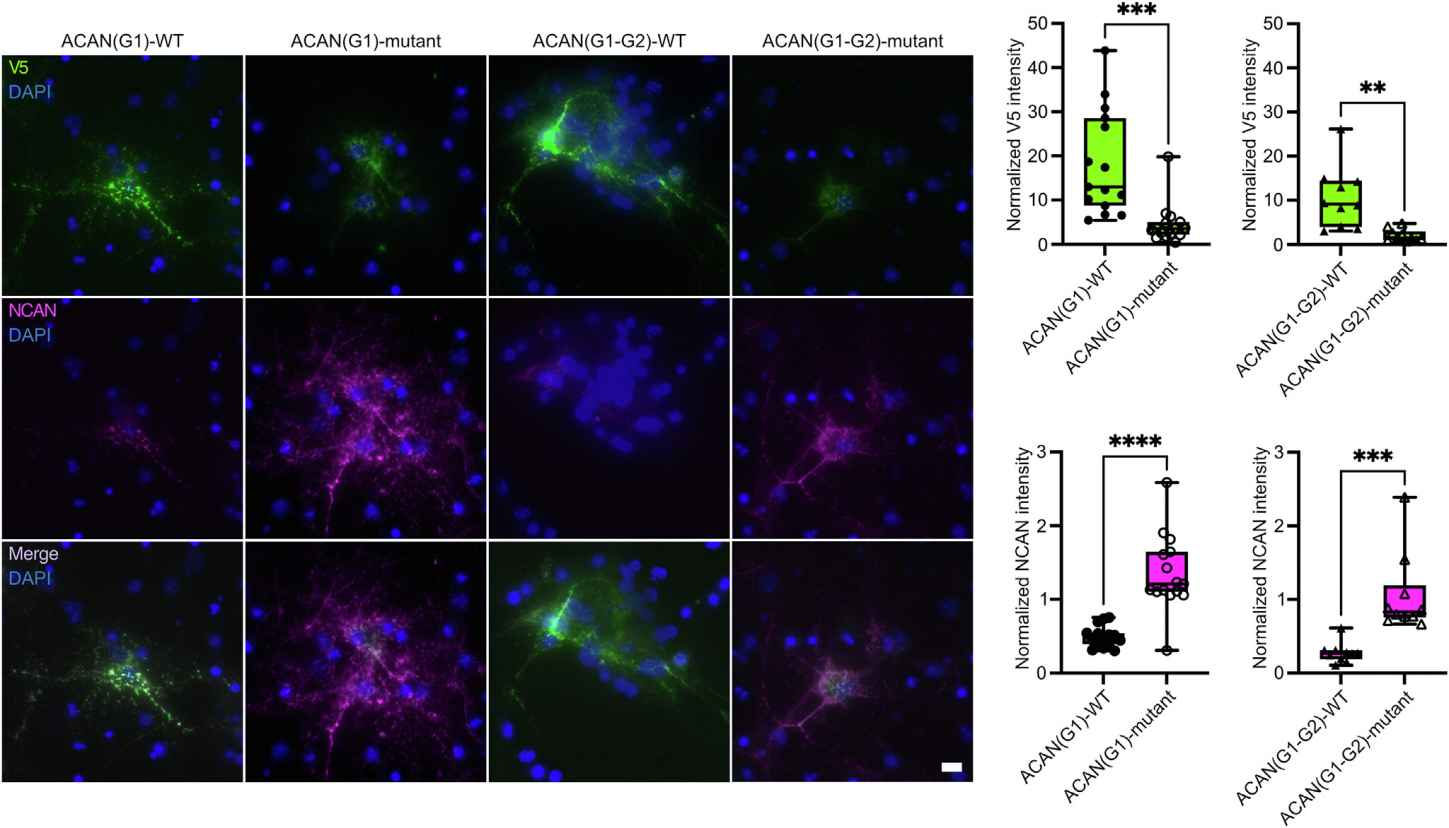
**Mutant forms of ACAN lacking HA-binding activity still immobilize specifically on PNN-bearing neurons.** Purified V5-tagged WT and HA-binding mutant forms of ACAN(G1), labeled ACAN(G1)-WT and ACAN(G1)-mutant, and ACAN(G1-G2), labeled ACAN(G1-G2)-WT and ACAN(G1-G2)-mutant, were exogenously added to primary neuronal cultures at a final concentration of 50 nM. The WT and mutant constructs all localized specifically to PNNs. However, the HA-binding mutants showed significantly lower V5 staining intensity (binding: ACAN(G1)-mutant 24.8%, *p* = 0.0002, ACAN(G1-G2)-mutant 19.6%, *p* = 0.0014). The impact of ACAN constructs on PNNs was assessed *via* colabeling with the endogenous PNN marker NCAN. Data were normalized to untreated control cells (a value of 1 representing equivalent NCAN staining to untreated controls). WT ACAN(G1) and ACAN(G1-G2) fragments disrupt NCAN staining at significantly higher levels than their mutant counterparts (NCAN intensity compared to HA-binding mutant: ACAN(G1)-WT 36.16%, *p* < 0.0001, ACAN(G1-G2)-WT 26.38%, *p* = 0.0003). The mutant constructs showed no disruption of NCAN staining in PNNs compared to untreated cells. Unpaired *t* test, N = 4 independent cultures, 3 to 5 PNNs images per culture per condition. The scale bar represents 10 μm. ACAN, aggrecan; DIV, day *in vitro*; NCAN, neurocan; PNN, perineuronal net.

We next compared the binding of the WT and HA-binding mutant of ACAN to PNNs using both the G1 and G1-G2 regions. Since it appeared that a concentration of 50 nM provided optimal binding for WT constructs, we utilized this concentration to assess differences in PNN binding between the WT and HA-binding mutant constructs. The intensity of binding of the HA-binding mutants to PNNs compared to WT constructs was significantly reduced by almost 5-fold (Fig. 8, ACAN(G1)-mutant: 24.8%, *p* = 0.0002, ACAN(G1-G2)-mutant: 19.6%, *p* = 0.0014). However, quite surprisingly, the HA-binding mutant constructs still bound specifically to PNNs, albeit with significantly lower intensity. Importantly, as with the WT constructs, concentrations above 50 nM did not increase PNN binding but instead led to nonspecific binding and aggregation (Fig. S10). At no concentration did the intensity of PNN binding of the HA-binding mutant constructs became equivalent to that of the WT constructs. These data suggest that ACAN can bind to net-bearing neurons specifically independently of its HA-binding activity.

Although the role ACAN plays in the formation of PNNs is still being defined, the sum of the available data suggests that it forms a molecular bridge in PNNs by binding HA *via* its N-terminus and the essential PNN component TNR *via* its C-terminus. Therefore, we hypothesized that exogenous addition of N-terminal fragments of ACAN would displace full-length endogenous ACAN, which would impair the molecular bridge to TNR and thus disrupt PNNs. To assess the impact of these constructs on PNNs, we analyzed the staining of the PNN component NCAN. In line with our hypothesis, the WT ACAN(G1) and ACAN(G1-G2) proteins disrupted NCAN staining in PNNs significantly at the 50 nM concentration (Fig. 8, ACAN(G1)-WT: 36.16%, *p* < 0.0001, ACAN(G1-G2)-WT: 26.38% of untreated control levels, *p* = 0.0003). In contrast, the variants of ACAN(G1) and ACAN(G1-G2) lacking HA-binding activity did not disrupt NCAN staining in PNNs. Therefore, although HA binding does not appear to be required for N-terminal constructs of ACAN to localize selectively to PNNs, it appears to play an important role in maintaining the integrity of PNNs at the neuronal surface.

## Discussion

PNNs were identified more than a century ago, yet it is only since the late 1990s that analyses of their molecular components and the molecular interactions that promote the assembly of nets have been undertaken. Although it is widely accepted that HA is abundant in PNNs (32, 46), the roles played by HA and protein–HA interactions in the assembly of nets are less clear-cut. For example, experiments aiming to reconstitute PNNs in HEK293 cells show that expression of HAS3 along with the essential net components ACAN and HAPLN1 promotes the formation of PNN-like structures around the cells (18). HAS3 is the HAS most frequently expressed in PNN-bearing cells (32), yet the number of neurons bearing nets is not reduced in the hippocampus or cortex of *Has3*^−/−^ mice (47). With these somewhat conflicting considerations in mind, it was of interest to analyze the importance of the HA-binding activity of ACAN to its integration into PNNs.

To achieve this objective, the crystal structure of the N-terminal G1 domain of ACAN was determined in the presence of a HA decasaccharide. These structural insights made it possible to engineer a variant of ACAN that does not associate with HA. The abilities of the G1 and G1-G2 domains ACAN to bind to PNN-bearing neurons from mouse cortical cultures were then tested at distinct concentrations. In these experiments, the G1 and G1-G2 regions behaved in an essentially identical fashion: they could both bind to net-bearing neurons and inhibit the integration of another PNN component, NCAN, into nets. Unexpectedly, however, mutant forms of ACAN(G1) and ACAN(G1-G2) that do not bind HA in BLI binding assays retained reduced but significant binding to net-bearing neurons compared to WT proteins. Thus, it appears that the selective insertion of aggrecan into PNNs is not solely dependent on its interaction with HA.

Our results strongly suggest that ACAN can assemble into nets through HA-dependent and HA-independent binding activities, but they do not shed light on the nature of the latter interactions in the recruitment of ACAN into PNNs. Because the recombinant proteins used in PNN-binding assays do not include chondroitin sulfate or keratan sulfate chains, mutant forms of ACAN are unlikely to be recruited into PNNs through glycosaminoglycan-binding proteins in net-bearing neurons (Fig. S11). In addition, both the G1 and G1-G2 regions of ACAN lacking HA-binding activities associated with nets (Fig. 8), so that the HA-independent recruitment of ACAN likely only depends on its G1 region. Finally, although nets are rich in chondroitin sulfate chains (33), the G1-G2 region of ACAN does not associate with glycosaminoglycan chains (14). As such, one possible hypothesis is that ACAN is recruited to nets *via* interactions with the ACAN-binding protein HAPLN1 since ACAN is associated with HAPLN1 through its G1 domain (15). HAPLN1, in turn, could be immobilized to the neuronal surface through interactions with HA synthesized by an HAS. Such a hypothesis would be consistent with results from the Fawcett lab indicating the expression of HAS3, HAPLN1, and ACAN in HEK293 cells was sufficient to induce the formation of a condensed matrix around the cells (18). Yet, even though the presence of PNNs is reduced in *Hapln1*-null mice, some residual ACAN still localizes to neurons bearing attenuated nets (17). Obviously, this result could be interpreted as ACAN immobilizing on neurons owing to its interactions with HA. However, at this stage, it is not possible to exclude that a yet undescribed protein may function as a receptor for ACAN on the surface of net-bearing neurons.

The potential ability of ACAN to assemble into PNNs because of protein–protein as well as protein–HA interactions would also explain a somewhat counterintuitive result. Net-like structures that include HAPLN1 and TNR persist in dissociated cortical cultures of cartilage matrix deficiency (cmd) mice (20). In cmd mice, a deletion of 7 base pairs in the gene encoding ACAN results in the expression of a truncated protein that includes the N-terminal Ig domain and part of the Link-1 module (9). Based on the results reported here, this truncated protein would be unable to bind HA. Homozygous mice carrying this mutation die soon after birth, preventing investigation of PNNs in their neural tissues. Apart from considering possible compensatory mechanisms to mitigate defective ACAN secretion (48), the presence of PNN-like structures in dissociated cortical cultures from cmd mice is difficult to explain, except if the truncated ACAN protein expressed by these cells is sufficient to induce condensation of the matrix around neurons, even if it is unable to bind HA. Thus, we hypothesize that the N-terminus of ACAN, and perhaps more specifically its N-terminal Ig domain, mediates protein–protein interactions that promote the formation of PNNs.

Another interesting question centers around the role of ACAN in PNNs. When PNNs were reconstituted on the surface of HEK293 cells, the expression of ACAN, HAPLN1, and HAS3 was necessary to obtain a condensed matrix around the cells (18). In contrast, the expression of HAPLN1 and HAS3 only resulted in a diffuse matrix. The condensed aspect of the matrix could stem from more significant cross-linking of HA chains through the combined interaction of ACAN with HA and HAPLN1. Viewed from that angle, it is troubling that the G2 domain of ACAN does not appear to bind HA in spite of the resemblance between its Link modules and those found in G1 because it could provide some of the additional cross-links needed to condense the matrix around neurons. Perhaps the G2 fragment binds to HA at concentrations well beyond those tested in our BLI assays, but these could be attained in the context of PNNs where the local respective concentrations of ACAN and HA are presumably much higher than in the assays performed here. Another possibility is that the G2 region binds to another protein, which induces changes in the structure of G2 to promote HA binding. However, in the context of dissociated neuronal cultures, the G2 domain of ACAN does not bind to PNNs (Fig. S12). Thus, at this stage, we remain unable to ascribe a role to the G2 region, and additional experiments focused on identifying its binding partners are needed. Finally, a structural argument could be made that it is only the binding of HA to ACAN(G1) that promotes condensation of the matrix. Indeed, the structure reported here indicates that the conformation of HA is altered when bound to ACAN (Fig. S3). This change in conformation in the bound HA may induce a local tension in the HA strand, which repeated over the length of several crisscrossing HA chains might trigger condensation of the matrix. These possible explanations for the condensation of the matrix around neurons are not mutually exclusive. As such, future characterization of the molecular interactions mediated by the distinct domains of ACAN will provide additional keys to understand why it is such an essential component of nets.

## Experimental procedures

### Cloning

A complementary DNA (cDNA) encoding the G1 region of human ACAN (UniProt ID P16112, amino acids 21–351) was synthesized by GenScript and cloned into a derivative of the pLex2 vector (49). This vector directs the expression of a signal peptide from bovine serum albumin followed by an hexahistidine tag, a human rhinovirus 3C protease site and ACAN(G1). A cDNA fragment encoding human ACAN(G2) or ACAN(G1) with the mutations R163A, R217A, R326A, and Q334A were generated by Integrated DNA Technology and cloned into the pLex2 derivative, as described above. Fragments encoding ACAN(G1) with the mutations D200E, G319S, and G219P+S298Y were synthesized by Twist Bioscience and cloned into the same expression plasmid.

A similar strategy was used to express the G1-G2 region of human ACAN. cDNA fragments encoding amino acids 21 to 676 of WT human ACAN or human ACAN including the changes R163A, R217A, R326A, and Q334A were synthesized by GenScript and cloned into a derivative of the pLex2 vector. In this case, however, this vector directs the expression of a signal peptide from human interleukin-2 followed by an hexahistidine tag, a human rhinovirus 3C protease site, ACAN(G1-G2), and a C-terminal V5 tag. Fragments encoding ACAN regions used in neuronal assays shown in Figures 7 and 8 were all cloned into this vector. All plasmid constructs were verified by DNA sequencing.

### Protein expression and purification

HEK293T/17 cells (ATCC # CRL-11268) used for transfection were grown in suspension in FreeStyle F17 media (Thermo Fisher Scientific) supplemented with 8 mM L-Glutamine and 1% (v/v) ultralow IgG fetal bovine serum (Gibco). Cells were maintained in vented polycarbonate flasks shaken at 135 rpm and kept at 37 °C and 8% CO_2_ (50). The day before transfection, cells were seeded at 8 × 10^5^ cells/ml in vented polycarbonate shaker flasks. On the day of the transfection, cells were counted and diluted 1 × 10^6^ cells/ml right before adding a solution of DNA–PEI complex. For transfecting a 100 ml culture at 1 × 10^6^ cells/ml, this solution includes 100 μg of plasmid mixed with 300 μl of a 1 mg/ml solution of PEI MAX (Polysciences) in 4 ml of OptiPRO SFM media (Thermo Fisher Scientific). The solution of DNA-PEI was incubated for 10 to 30 min at room temperature before being added to the cells. The cells were maintained at 37 °C for ∼12 to 16 h, then switched to a temperature of 33 °C. Cultures were harvested 5 to 6 days after. Kifunensine (Carbosynth) was added to a final concentration of 7.5 μM to cells the day of the transfection to generate ACAN(G1) with oligomannose N-linked carbohydrates that can be removed by endoglycosidases prior to initiating crystallization trials (51).

Transfected cells and conditioned media were separated by centrifugation and conditioned media was supplemented with solutions of 400 mM sodium phosphate pH 7.5 and 5 M NaCl to final concentrations of 40 mM and 500 mM, respectively. The media was then applied to a 5-ml HisTrap excel column (Cytiva) equilibrated in 500 mM NaCl, 50 mM sodium phosphate pH 7.5. The bound protein was eluted with a gradient to 500 mM imidazole over 15 column volumes. Rhinovirus 3C protease or a mixture of human rhinovirus 3C protease, endoglycosidase H, and endoglycosidase F were added to the purified protein and dialyzed overnight at room temperature against 250 mM NaCl, 50 mM sodium phosphate, pH 7.5, 40 mM imidazole, and passed over a 5-ml His-Trap column (Cytiva). The flow through was kept. Subsequent purification steps involved ion exchange on a 5-ml HiTrap Q HP column (Cytiva) equilibrated in 20 mM Tris–HCl pH 8.0, followed by gel filtration chromatography on a Superdex 75 26/600 column (Cytiva) equilibrated in 150 mM NaCl and 20 mM Na-Hepes pH 7.5.

The structural integrity of ACAN(G1) with the mutations R163A, R217A, R326A, and Q334A was evaluated using CD. Samples of purified WT ACAN(G1) and the site-directed mutant were prepared in 10 mM potassium phosphate (pH 7.5) to a final concentration of 0.2 mg/ml. Spectra were recorded from 250 to 190 nm in scanning mode using a Jasco J-1500 spectropolarimeter, with a 1 nm bandwidth and a scan rate of 100 nm/min. A total of three replicates were collected and averaged using software provided by the manufacturer. Because these spectra were not collected under a vacuum, all data from wavelength values less than 200 nm should be considered with caution.

For PNN-integration assays, constructs expressing V5-tagged ACAN fragments were transfected into adherent HEK293 cells using a PEI transfection method. Briefly, for each 100 mm plate of HEK293 cells, 10 μg of DNA construct was added to 1 ml of serum-free Dulbecco's modified Eagle's medium (Thermo Fisher Scientific). A 21-μl aliquot of PEI at 1 mg/ml was added to the DNA solution and incubated at room temperature for 15 min. The transfection mix was added to a 100-mm plate at ∼ 80% confluency. Number of plates and volumes were scaled as required. Cells were switched to serum-free Opti-MEM media (Thermo Fisher Scientific) 24 h after transfection. Conditioned media from transfected HEK293 cells were collected 48 h posttransfection and concentrated using 30,000 MWCO concentrators (Amicon Ultra, EMD Millipore). Protein of interest from the concentrated media was purified using Cobalt Spin Columns (Thermo Fisher Scientific). Samples were mixed with wash buffer (50 mM sodium phosphate, 300 mM NaCl, 10 mM imidazole; pH 7.4) and applied to the column. Columns containing samples were incubated for at least 30 min at 4 °C in an orbital or end over end shaker. Columns were washed three times with wash buffer and protein was eluted using 50 mM sodium phosphate, 300 mM NaCl, 150 mM imidazole; pH 7.4.

For addition to neurons, eluted protein from above was desalted using Zeba spin desalting columns (Thermo Fisher Scientific) as per the manufacturer’s protocol and concentrated using Pierce protein concentrators (Thermo Fisher Scientific). Samples were diluted to 1 μM in Neurobasal media (Thermo Fisher Scientific) and added to cells as required.

### Crystallization, structure determination, and structural analyses

Crystals were grown at 20 °C by hanging drop vapor diffusion. For crystallization of ACAN(G1), a 1-μl aliquot of deglycosylated protein at 7.4 mg/ml in 10 mM Hepes pH 7.5, 75 mM NaCl was mixed with 1 μl of 1% (w/v) PEG 3,350, 100 mM Bis-Tris, pH 5.5, and 600 mM ammonium sulfate. Crystals were frozen in mother liquor supplemented with 30% (v/v) glycerol. For crystallization of the ACAN(G1)–HA complex, a 1-μl aliquot of deglycosylated protein at 7.4 mg/ml (∼200 μM) in 10 mM Hepes pH 7.5, 75 mM NaCl was mixed with 1 μl of a 1 mM solution of HA decasaccharide in water (HA_10_, Iduron, distributed by Galen Laboratory Supplies) and 1 μl of 15% (w/v) PEG 10,000, 50 mM Bis-Tris propane, pH 9.0, and 200 mM ammonium acetate. Crystals were frozen in mother liquor supplemented with 20% (v/v) PEG200 and 50 μM of HA_10_.

X-ray diffraction data were collected on beamline 22-ID of the Advanced Photon Source at Argonne National Laboratory. Diffraction data were processed using HKL2000 (52). Structures were determined by molecular replacement in PHASER as implemented in PHENIX (53, 54) using a model of human ACAN(G1) generated with AlphaFold2 (34). For each model, an initial rebuilding was undertaken using PHENIX.AUTOBUILD followed by iterative cycles of model building in COOT (https://www2.mrc-lmb.cam.ac.uk/personal/pemsley/coot/) (55) and positional, individual B-factor, and translation-libration-screw (TLS) refinement using PHENIX.REFINE. Secondary structure restraints were used in the case of the ACAN(G1) crystallized in the absence of HA. The final models were validated using the RSCB PDB validation server. Lists of interacting residues and buried surface area were generated with Chimera X (56). RMSD values between superimposed structures were calculated with GESAMT (57) implemented in CCP4 (58). For structural comparisons, structures were superimposed using the least-square or secondary structure matching options in COOT. All structural representations were generated using ChimeraX.

### HA-binding assays using BLI

Interactions between fragments of human ACAN and HA were quantified at room temperature in 150 mM NaCl, 10 mM Na-Hepes pH 7.5, 1 mg/ml bovine serum albumin, 0.02%(v/v) Tween-20 using an Octet K2 system (Sartorius). Fragments of HA with a single biotin molecule attached at the reducing end were purchased from Creative PEGWorks. Monobiotin HA chains (250 nM for 20-kDA HA chains and 25 pM for 1-MDa HA chains) were immobilized onto streptavidin tips (Sartorius). The signals for the loading of biotinylated HA at these two distinct concentrations onto streptavidin sensors were comparable (∼0.7 nm). These tips were then incubated with various concentrations of purified ACAN proteins for 90 to 180 s during the association phase and then in buffer only during the dissociation phase for 90 to 180 s. The signal was corrected by subtracting the background measured for the buffer only. Experimental data were analyzed using a 1:1 binding model, as implemented in the Octet Data Analysis software (version 11.0.0.4; https://www.sartorius.com). For visualization, sensorgrams were plotted using Prism 10 (GraphPad Software; https://www.graphpad.com). The results are reported as the average of at least four replicates. These include at least two independent measurements for each of at least two distinct batches of proteins. Additional information about individual experiments used in affinity calculations is listed in Table S1.

### Sequence alignments

Amino acid sequences for aggrecan for human (UniProt ID P16112), mouse (UniProt ID Q61282), chicken (UniProt ID P07898), and zebrafish (UniProt ID F1QDA1 for Acana and UniProt ID A0A8M9P6I2 for Acanb) were aligned using Clustal Omega available from the European Bioinformatics Institute website (59).

### Animals

For primary cortical neuronal cultures, timed pregnant CD-1 WT mice were purchased from Charles River Laboratories. All experiments followed the protocols approved by the Institutional Animal Care and Use Committee of SUNY- Upstate Medical University.

### Primary cortical cultures

Primary cortical neuronal cultures were prepared as described previously (20, 60). Briefly, cortices from embryonic day (E) 16 CD-1 WT embryos were dissected out and digested with 0.25% trypsin-EDTA (Thermo Fisher Scientific) for 25 min. The tissue ball after trypsin digestion was treated with RNA-ase free DNA-ase (Promega) for 6 min and passed through a 70-μm cell strainer (Falcon). Cells were centrifuged to remove any residual DNA-ase and resuspended in Neurobasal medium supplemented with B27, GlutaMAX, and penicillin-streptomycin (Thermo Fisher Scientific). Cultures were plated at a density of 2.1 × 10^6^ cells/ml on glass coverslips precoated with poly-D-lysine (100 μg/ml, Sigma-Aldrich) and laminin (50 μg/ml, Thermo Fisher Scientific). Cells were treated with 5 μM cytosine arabinoside (ASigma-Aldrich) from 1 to 3 days *in vitro* (DIV) to eliminate glia. Culture media was replaced at 3 DIV after arabinoside treatment, followed by a half-media change at 6 DIV. For PNN integration experiments, purified ACAN fragments tagged with V5 (see above) were added to cells at 6 DIV as required. Cells were maintained at 37 °C, 5% CO_2_ until fixation at 9 DIV.

### Immunocytochemistry and Western blot analyses

Primary cortical cultures plated on coverslips were fixed in cold 4% phosphate-buffered paraformaldehyde with 0.01% glutaraldehyde, pH 7.4 at 9 DIV. Afterward, the cells were blocked in screening medium (Dulbecco's modified Eagle's medium, 10% (v/v) fetal bovine serum, 0.2% (w/v) sodium azide) for 1 h, before adding primary antibodies overnight at 4 °C (rabbit anti-V5: invitrogen MA5-32053; sheep anti-NCAN: biotechne AF5800). The next day, Alexa Fluor-conjugated secondary antibodies (ThermoFisher Scientific) in screening medium were added to the cells for 2 h before mounting the coverslips with ProLong Antifade Kit (Thermo Fisher Scientific). Cell nuclei were visualized with Hoechst solution (Thermo Fisher Scientific) diluted in PBS. For quantification of probe binding, large-tiled images (1259 μm × 941 μm) were taken using a 20× objective (NA = 0.8). Images were converted to 8 bit and background correction was carried out by subtracting modal value from images. The intensity of binding was determined using the measure function in ImageJ (https://imagej.net/). For NCAN disruption experiments, individual PNNs were imaged using a 40× oil objective (NA = 1.4). Background subtraction was carried out using the rolling ball background correction function in ImageJ. The intensity of NCAN in PNNs was determined in ImageJ using the measure function.

The potential presence of chondroitin sulfate and keratan sulfate chains on the G1 and G1-G2 fragments of ACAN used in neuronal cultures was assessed using chondroitinase ABC and keratanase I. V5-tagged WT and HA-binding mutant fragments of ACAN comprising the G1 domain or the G1-G2 domains were expressed in HEK293 cells, collected from the media and concentrated. Proteins (100 μg) were digested with 0.01 units of chondroitinase ABC (Sigma-Aldrich, C3667) or 0.05 units of keratanase 1 (R&D systems, 8620-GH) for 4 h at 37 °C. Protein aliquots (15 μg per lane) were loaded of an 8% SDS polyacrylamide gel and transferred to a 0.45 μm nitrocellulose membrane. Bands were detected with an anti-V5 rabbit mAb and imaged using Cytiva ImageQuant 800.

## Data availability

The atomic coordinates and structure factors (codes 9DFT, 9DFF) have been deposited in the Protein Data Bank (https://www.rcsb.org). All other experimental data that are not contained within the article or the supporting information are available upon request by contacting the corresponding authors R. T. M. (matthewr@upstate.edu) or S. B. (bouyains@umkc.edu).

## Supporting information

This article contains supporting information.

## Conflict of interest

The authors declare that they have no conflicts of interest with the contents of this article.
