# Supplementary material for: Aggrecan immobilizes to perineuronal nets through hyaluronan-dependent and hyaluronan-independent binding activities[image]
Source: J Biol Chem. 2025 Apr 22;301(6):108525. doi: 10.1016/j.jbc.2025.108525 (PMC12155565; doi:10.1016/j.jbc.2025.108525)
Supplement: Supplementary information [file mmc1.pdf]

Supporting information for:

Aggrecan immobilizes to perineuronal nets through hyaluronan-dependent and independent binding activities

Matthew Y. Otsuka[1], Leslie B. Essel[1,#], Ashis Sinha[2], Gabrielle Nickerson[2], Seth M. Mejia[2], Ashley Edge[1], Russell T. Matthews[2,*], Samuel Bouyain[1,*]

From the [1]Division of Biological and Biomedical Systems, School of Science and Engineering, University of Missouri-Kansas City, Kansas City, Missouri 64110 and the [2]Department of Neuroscience and Physiology, State University of New York Upstate Medical University, Syracuse, New York 13210

***corresponding authors:** Russell T. Matthews, matthewr@upstate.edu; Samuel Bouyain, bouyains@umkc.edu.

Supporting information includes:

- Table S1 – Excel spreadsheet, available separately
- Fig. S1, included in this file
- Fig. S2, included in this file
- Fig. S3, included in this file
- Fig. S4, included in this file
- Fig. S5, included in this file
- Fig. S6, included in this file
- Fig. S7, included in this file
- Fig. S8, included in this file
- Fig. S9, included in this file
- Fig. S10, included in this file
- Fig. S11, included in this file
- Fig. S12, included in this file

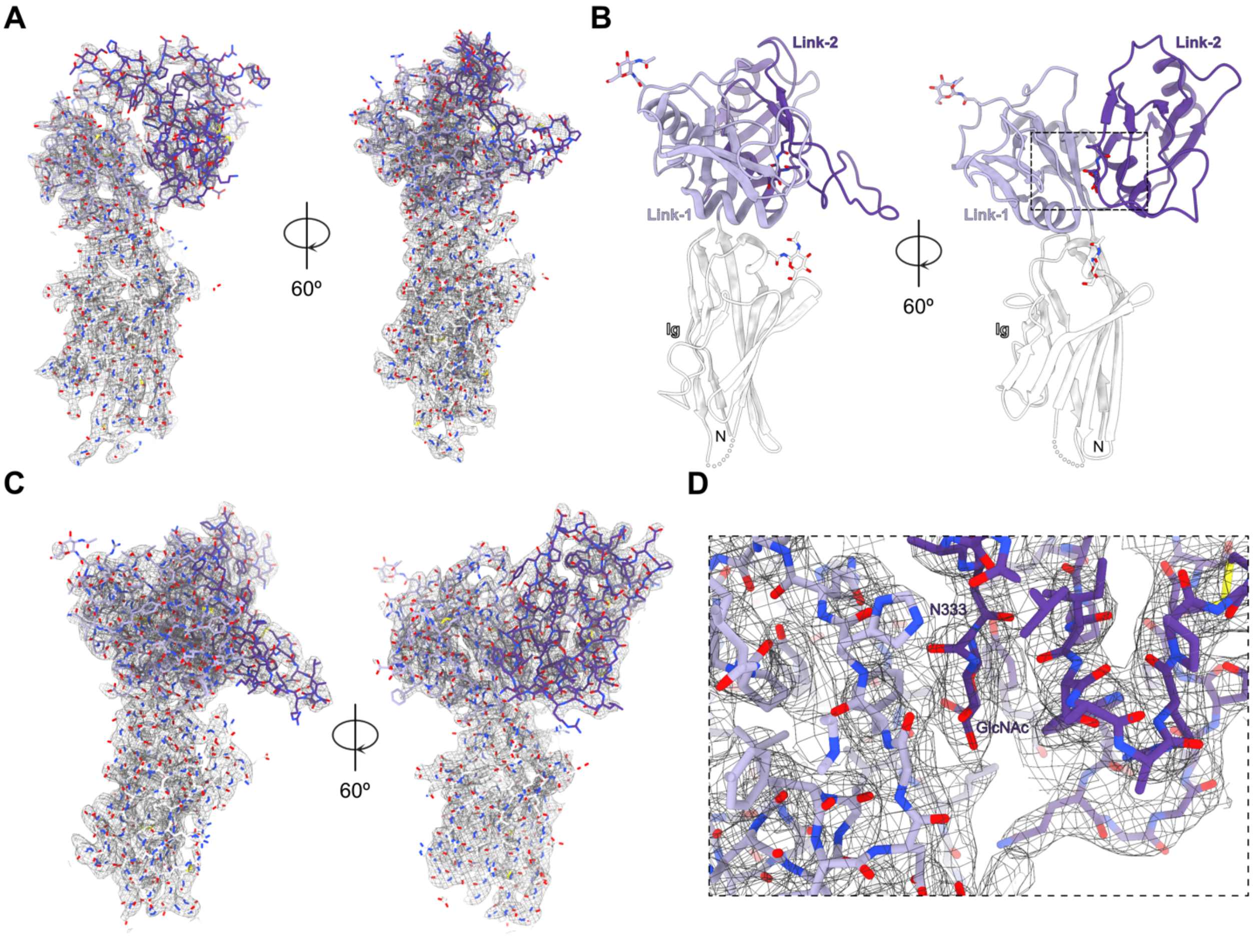

Figure S1. Electron density maps for the two chains of ACAN found in the asymmetric unit of the ACAN(G1) crystal structure.

A. 2mFo-DFc electron density map for chain B of ACAN(G1) at 3.5 Å shown in Fig. 1 is contoured at 1.2 σ. ACAN(G1) is shown as sticks and the Ig, Link-1, and Link-2 domains are colored white, lilac, and violet, respectively. The two views are in the same orientation as the views shown in Fig. 1B.
B. The G1 region of chain A of human ACAN is shown in a ribbon diagram in two distinct orientations related by a 60º rotation. The Ig, Link-1, and Link-2 domains in chain B of ACAN are colored white, lilac, and violet, respectively. The letters N and C indicate the N- and C-termini, respectively. Asparagine-linked N-acetylglucosamine residues are shown as sticks along with the asparagine side chain. A disordered region in the Ig domain is shown as a dotted line. An area boxed by a dotted line shows the GlcNAc residue on N333. This region is shown in more detail in panel D.
C. 2mFo-DFc electron density map for chain A of ACAN(G1) at 3.5 Å shown in panel B is contoured at 1.2 σ. ACAN(G1) is shown as sticks and the Ig, Link-1, and Link-2 domains are colored white, lilac, and violet, respectively. The two views are in the same orientation as the views shown in panel B.
D. 2mFo-DFc electron density map for the region surrounding N333 in chain A of ACAN(G1) at 3.5 Å shown in panel B is contoured at 1.2 σ.

ACAN, aggrecan; GlcNAc, *N*-acetylglucosamine; Ig, immunoglobulin;

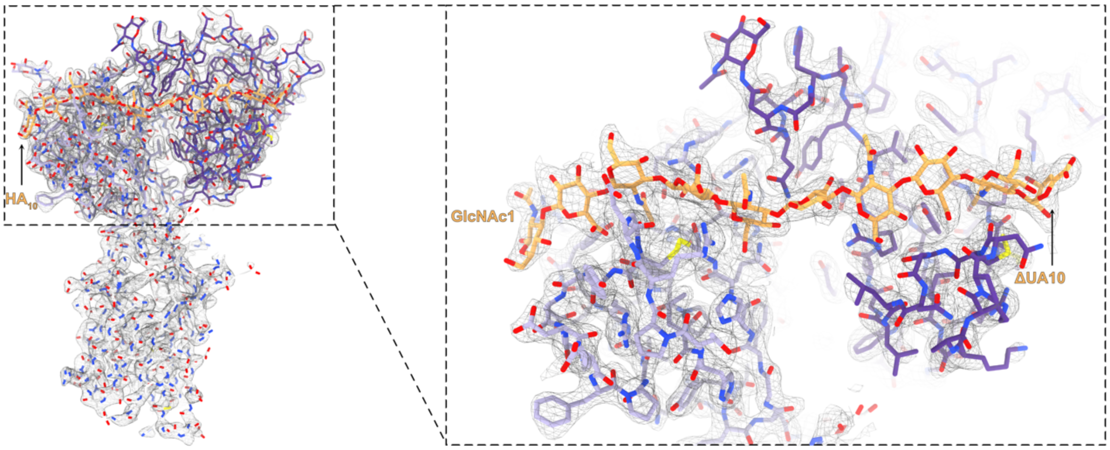

Figure S2. Electron density map for the ACAN-HA$_{10}$ complex. Representative electron density maps at the interface.
The ACAN-HA$_{10}$ complex is shown here in stick representation along with a 2mFo-DFc electron density map contoured at 1.2 σ. The Ig, Link-1, and Link-2 domains of ACAN are colored white, lilac, and violet, respectively. The bound glycosaminoglycan is in orange. The boxed inset shows a close-up view of the HA-binding site in ACAN.
ACAN, aggrecan; GlcNAc, *N*-acetylglucosamine; HA, hyaluronan; ΔUA, 4-deoxy-alpha-L-threo-hex-4-enopyranuronic acid.

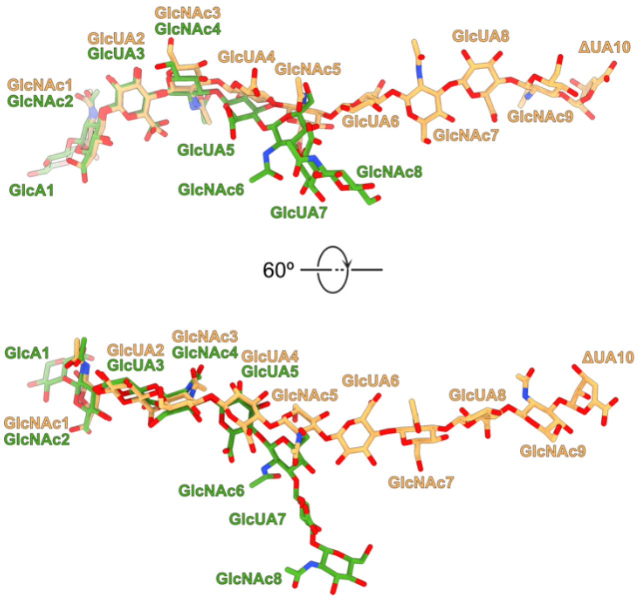

Figure S3. Comparison of HA oligosaccharides from powdered diffraction and in the crystal structure of the ACAN-HA complex.
The HA oligosaccharide bound to ACAN is shown here as orange sticks. A structure of a HA octasaccharide determined in the absence of bound protein (PDB ID 3HYA) is superimposed onto HA_{10} using residues GlcNAc1 of HA_{10} and GlcNAc2 of “free” HA. In the top view, the HA_{10} oligosaccharide is in the same orientation as the one shown in Fig. 2A.
ACAN, aggrecan; GlcNAc, *N*-acetylglucosamine; GlcA, alpha-D-glucopyranuronic acid; GlcUA, beta-D-glucopyranuronic acid; HA, hyaluronan; ΔUA, 4-deoxy-alpha-L-threo-hex-4-enopyranuronic acid.

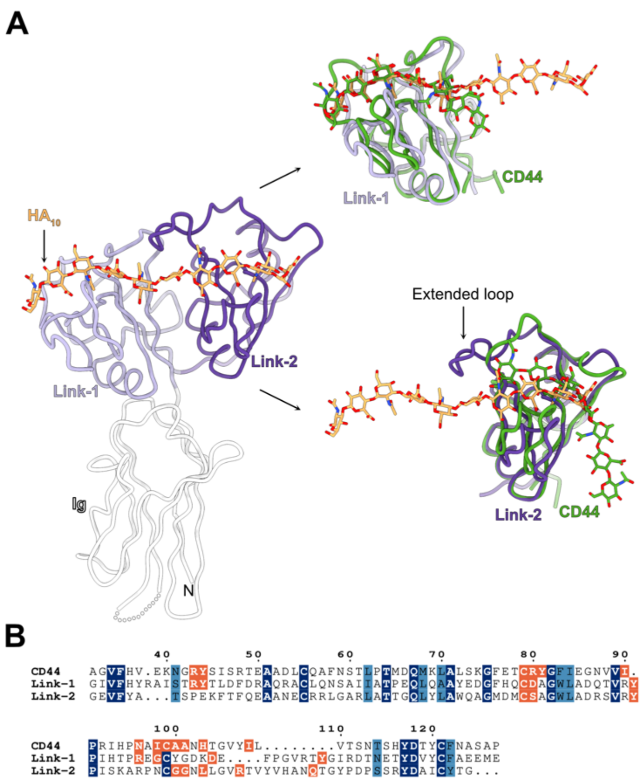

Figure S4. Comparison of CD44-HA complex with the HA_{10}-bound Link-1 and Link-2 domains of human ACAN.

A. The bound HA_{10} oligosaccharide in ACAN occupies grooves in the Link-1 and Link-2 modules of ACAN that match the site where HA binds to the Link module in mouse CD44 (PDB ID 2JCR). Proteins are shown in coil representation, while the bound oligosaccharides are shown as sticks. The Link module of mouse CD44 was superimposed onto the two Link modules of ACAN and colored green. The Ig, Link-1, and Link-2 domains in ACAN are colored white, lilac, and violet, respectively. The HA oligosaccharide bound to CD44 is also colored green. A loop extends from Link-2 to bind to HA. This loop is absent in CD44 and corresponds to an insertion of 8 amino acids between residues 109 and 110 of CD44.
B. Amino acid conservation between the Link module of mouse CD44 and the Link-1 and Link-2 modules of human ACAN. The numbering corresponds to mouse CD44 (Uniprot ID# P15379-14). Identical residues are shaded in navy, while similar residues are shaded in blue. Residues that interact with HA are shaded orange.

ACAN, aggrecan; HA, hyaluronan; Ig, immunoglobulin.

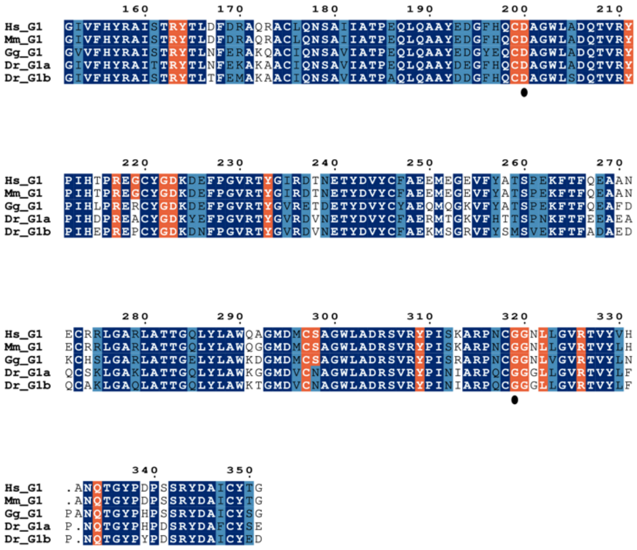

Figure S5. Sequence alignment of tandem Link modules found in the G1 domains of selected ACAN proteins.

Amino acid conservation between the tandem Link modules of human, mouse, chicken, and zebrafish ACAN. Two genes, *acana* and *acanb*, encode homologs of human ACAN in zebrafish. The numbering corresponds to human ACAN. Identical residues are shaded in navy, while similar residues are shaded in blue. Residues that interact with HA in human ACAN are shaded orange. They are highlighted in the same color in other sequences to indicate residue conservation. Black dots under D200 and G319 indicate residues that have been identified in patients suffering from osteochondritis dissecans and reported to the ClinVar database. ACAN, aggrecan; Hs, *Homo sapiens*; Mm, *Mus musculus*; Gg, *Gallus gallus*, Dr, *Danio rerio*.

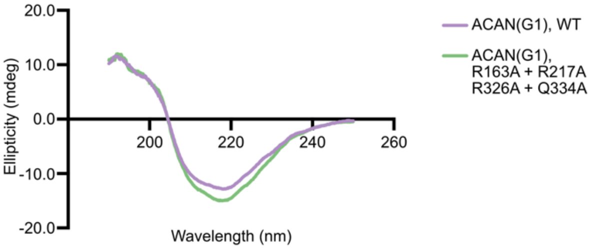

Figure S6. Circular dichroism analysis of wild-type ACAN(G1) and the site directed mutant R163A + R217A + R326A+ Q334A.
Purified wild-type and mutant human ACAN(G1) were dialyzed extensively against 10 mM potassium phosphate pH 7.5. Spectra were measured at concentrations of 0.2 mg/ml. Comparison of the two spectra indicates that the spectral features of the proteins are overall identical, and the differences observed for the two proteins are explained by differences in the protein concentration. Overall, these experiments indicate that the mutations do not affect the secondary structure of ACAN(G1).
ACAN, aggrecan

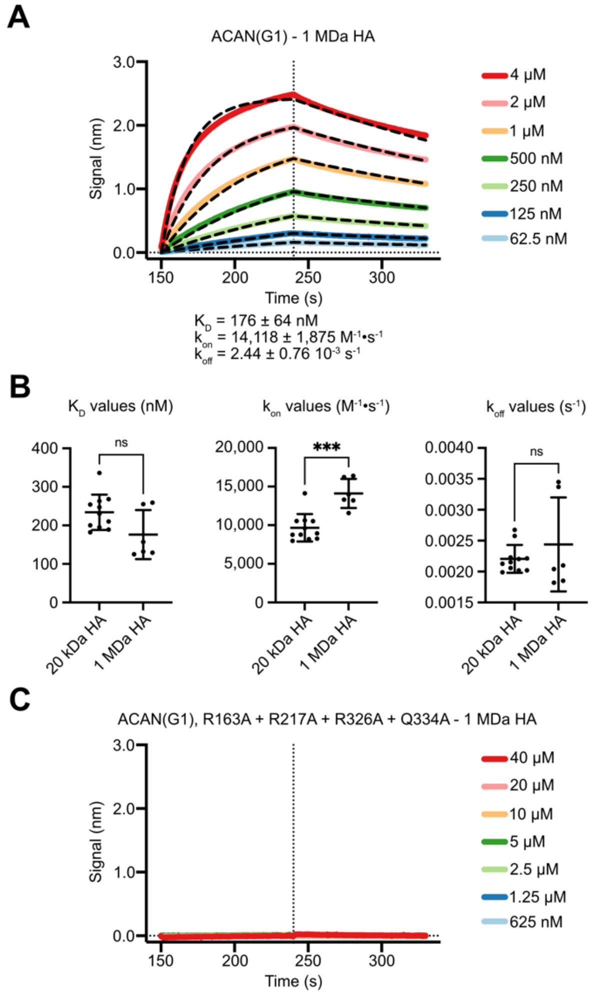

Figure S7. Characterization of ACAN(G1) binding to immobilized 1-MDa HA by biolayer interferometry.

A. Binding of the G1 domain of human ACAN to 1-MDa HA immobilized onto streptavidin sensors. Representative association and dissociation curves are shown here along with a vertical dashed line that indicates the start of the dissociation phase. Raw binding data are shown in distinct colors and were analyzed using a 1:1 binding model. Fitted binding curves are shown as black dashed lines. Values for the affinity constant (K_D), the rate of association (k_{on}), and the rate of dissociation (k_{off}) are reported as average ± standard deviation from six independent experiments using three distinct biological replicates. Additional information about individual experiments used in affinity calculations is listed in Table S1.

B. The differences between the values of K_D, k_{on}, and k_{off} for the interactions of ACAN(G1) with 20-kDa HA (11 independent experiments using 3 distinct protein batches) and 1-MDa HA (6 independent experiments using 3 distinct protein batches) were analyzed using a Welch t-test. The differences between the k_{on} values were statistically significant ($p=0.0008$), whereas the differences between K_D values and the k_{off} values were not.

C. Binding of the G1 domain of human ACAN including mutations to alanine at positions R163, R217, R326, and Q334 to 1-MDa HA immobilized onto streptavidin sensors. Representative association and dissociation curves are shown here along with a vertical

dashed line that indicates the start of the dissociation phase. The raw binding data did not show any interaction between immobilized HA and this variant of ACAN(G1).

ACAN, aggrecan; HA, hyaluronan

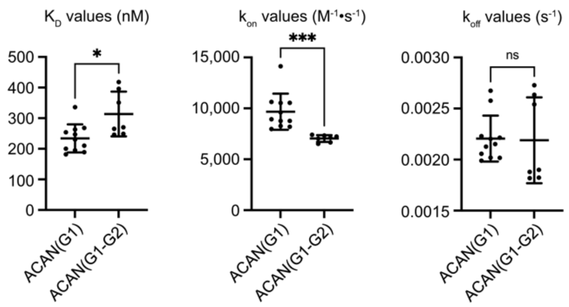

Figure S8. Statistical analyses of K_D, k_{on}, and k_{off} values measured for ACAN(G1) and ACAN(G1-G2).

The differences between the values of K_D, k_{on}, and k_{off} for the interactions of ACAN(G1) (11 independent experiments using 3 distinct protein batches) and ACAN(G1-G2) (7 independent experiments using 3 distinct protein batches) and biotinylated HA were analyzed using a Welch t-test. The differences between the K_D values were significant ($p = 0.0299$), as were those between the k_{on} values ($p = 0.0006$). The values for the off-rates were not statistically significant. ACAN, aggrecan

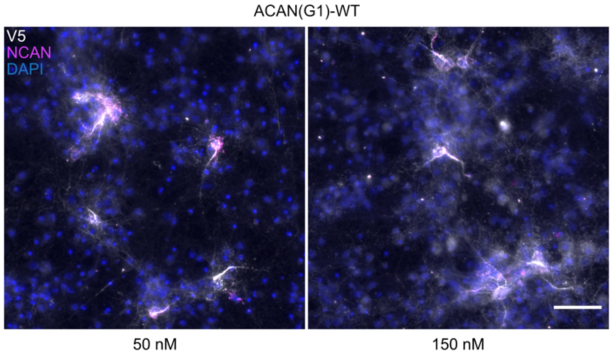

Figure S9. Additions of wild-type ACAN(G1) above 50 nM show increased non-specific binding on cultured neurons.
Purified wild-type (ACAN(G1)-WT) tagged with V5 was added at 6 DIV to cortical neurons at 50 nM or 150 nM. Cells were fixed and analyzed at 9 DIV. ACAN(G1)-WT, when tested at a concentration of 50 nM, bound specifically to PNNs as demonstrated by the colocalization of V5 reactivity with PNN marker NCAN on cultured cortical neurons. However, at a concentration of 150 nM or above, there was no change in the intensity of binding to PNNs but increased non-specific binding beyond PNNs. Therefore, we restricted our analyses to concentrations of 50 nM or lower. Scale bar 50 µM.
ACAN, aggrecan; DIV, day *in vitro*; NCAN, neurocan; PNN, perineuronal net

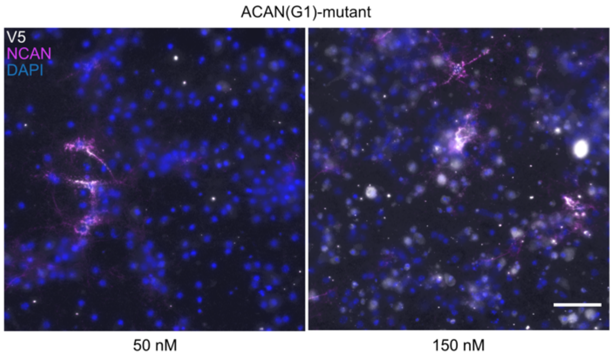

Figure S10. Additions of the HA-binding mutant of ACAN(G1) above 50 nM show increased non-specific binding on cultured neurons.
Purified HA-binding mutant of ACAN(G1) tagged with V5 was added at 6 DIV to cortical neurons at 50 nM or 150 nM. Cells were fixed and analyzed at 9 DIV. ACAN(G1)-mutant, when tested at a concentration of 50 nM, bound specifically to PNNs as demonstrated by the colocalization of V5 reactivity with PNN marker NCAN on cultured cortical neurons. However, at a concentration of 150 nM or above, there was no change in the intensity of binding to PNNs but we detected an increase in the formation of aggregates and non-specific binding. Therefore, there is no amount of ACAN(G1)-mutant that can be added that matches the binding intensity of the ACAN(G1)-WT. Scale bar 50 μM.
ACAN, aggrecan; DIV, day *in vitro*; NCAN, neurocan; PNN, perineuronal net

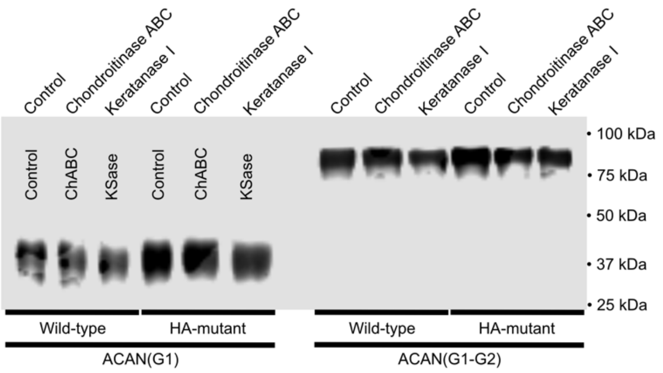

Figure S11. ACAN constructs produced in HEK293 cells are not modified by chondroitin or keratan sulfate glycosaminoglycan chains.
V5-tagged wild-type and HA-binding mutant fragments of ACAN comprising the G1 domain or the G1-G2 domains were expressed in HEK293 cells, collected from the media and concentrated. The proteins were treated with chondroitinase ABC or keratanase I and analyzed by Western blotting against V5. Digestion with either chondroitinase ABC or keratanase I had no discernible impact on the apparent molecular weight of bands, suggesting these constructs are not glycosylated with chondroitin sulfate or keratan sulfate chains when produced recombinantly in HEK293 cells.

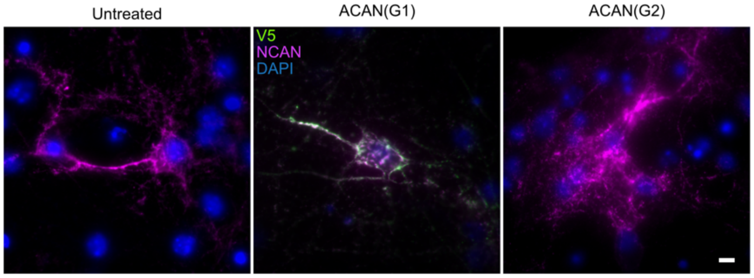

Figure S12. The ACAN G2 domain does not bind to PNN in cultured neurons. ACAN constructs comprising the G1 domain, ACAN(G1), and the G2 domain, ACAN(G2), were generated in HEK293 cells and purified from the media by cobalt affinity chromatography. They were added at a concentration of 5 nM to primary neuronal cultures at DIV 6. Cells were fixed at 9 DIV and stained for the PNN marker NCAN (magenta), V5 to detect the V5-tagged ACAN constructs (green) and nuclei were stained with DAPI (blue). ACAN(G1) bound with high specificity to PNNs in contrast with ACAN(G2), which did not associate with PNNs. Scale bar 10μM.
ACAN, aggrecan; DIV, day *in vitro*; NCAN, neurocan; PNN, perineuronal net
